# Nuclear transport of cancer extracellular vesicle-derived biomaterials through nuclear envelope invagination-associated late endosomes

**DOI:** 10.18632/oncotarget.14804

**Published:** 2017-01-24

**Authors:** Germana Rappa, Mark F. Santos, Toni M. Green, Jana Karbanová, Justin Hassler, Yongsheng Bai, Sanford H. Barsky, Denis Corbeil, Aurelio Lorico

**Affiliations:** ^1^ Roseman Cancer Center and Department of Pathology, Roseman University College of Medicine, Las Vegas, NV, USA; ^2^ Biotechnology Center and DFG Research Center, Technische Universität Dresden, Tatzberg, Dresden, Germany; ^3^ Indiana State University, Terre Haute, IN, USA; ^4^ Co-first author

**Keywords:** cancer, endosome, extracellular vesicle, mesenchymal stromal cell, nuclear envelope invagination

## Abstract

Extracellular membrane vesicles (EVs) function as vehicles of intercellular communication, but how the biomaterials they carry reach the target site in recipient cells is an open question. We report that subdomains of Rab7^+^ late endosomes and nuclear envelope invaginations come together to create a sub-nuclear compartment, where biomaterials associated with CD9^+^ EVs are delivered. EV-derived biomaterials were also found in the nuclei of host cells. The inhibition of nuclear import and export pathways abrogated the nuclear localization of EV-derived biomaterials or led to their accumulation therein, respectively, suggesting that their translocation is dependent on nuclear pores. Nuclear envelope invagination-associated late endosomes were observed in *ex vivo* biopsies in both breast carcinoma and associated stromal cells. The transcriptome of stromal cells exposed to cancer cell-derived CD9^+^ EVs revealed that the regulation of eleven genes, notably those involved in inflammation, relies on the nuclear translocation of EV-derived biomaterials. Our findings uncover a new cellular pathway used by EVs to reach nuclear compartment.

## INTRODUCTION

Extracellular membrane vesicles (EVs) are nanobiological units released from most cell types into the extracellular milieu. Depending on their biogenesis, they are referred to as exosomes and ectosomes [1, 2]. The former are released when the multi-vesicular bodies fuse with the plasma membrane, whereas the latter bud from the plasma membrane. Given the difficulty to isolate pure populations, we will refer to them collectively as EVs. The physiological functions of EVs are diverse, e.g., cell-cell communication, cellular differentiation, immunity and inflammation [3–5]. In cancerous tissues, the biomaterials carried by cancer cell-derived EVs can play a role in the development of the pre-metastatic niche. Therein, EVs can modulate the function of stromal components, notably multipotent mesenchymal stromal cells (MSCs), to favor cancer progression and metastasis [3, 6–8]. The EV-mediated crosstalk between cancer and non-cancerous cells can also be reciprocal [9]. Several key proteins and nucleic acids (e.g., mRNAs and microRNAs) in EVs modulate and/or interfere with the immunologic properties of MSCs and the interaction of cancer cell-derived EVs with B cells fosters tumor-promoting humoral immunity, ultimately playing a fundamental role in cancer progression [6, 10–12]. Lyden's group demonstrated that EVs promote organ-specific metastasis through their uptake into resident normal cells at their predicted metastatic destination [13].

Various mechanisms to explain the cellular uptake of EVs have been described [14]. The endocytic membrane transport pathway involving early and late endosomes is implicated in directing membrane-bound molecules into lysosomes or shuttling them back to the plasma membrane, directly or via the endoplasmic reticulum (ER) [14–16]. Despite this knowledge, the molecular fate of EV content upon their internalization by recipient cells remains elusive. This is particularly true for EV-associated proteins and nucleic acids that are shuttling to the nucleus of recipient cells [1, 17–19]. The presence of EV-derived biomaterials in the nuclei of host cells suggests that the nuclear pores might participate in the translocation of extracellular signals. Although nuclear envelope is generally viewed as a smoothly convex surface, frequent irregularities and tubular membrane-bound invaginations, sometimes branching ones, are reported in normal and abnormal cells found in animal and plant kingdoms [20]. Nuclear envelope invaginations are abundant in cancer cells and are of diagnostic and prognostic significance [21]. Thus, nuclear envelope morphology is an important component of tumor grade, presumably because nuclear envelope components have central roles in tumor development and progression, and its alteration has adverse prognostic significance in several types of cancer, including breast carcinoma [22]. The exact role of nuclear envelope invaginations is still not completely recognized. They might extend the functions of the peripheral nuclear envelope into the deep part of the nucleoplasm, providing additional spatial control within the nucleus. Numerous vesicular structures were observed in nuclear envelope invaginations [21].

In the present study, we have addressed the following question: do nuclear envelope invaginations participate in the transfer of extracellular signals carried by EVs to the nucleus of recipient cells? This issue was investigated using normal and cancer cell-derived EVs and, as recipient cells, human primary MSCs, FEMX-I malignant melanoma cells or MDA-MB-231 (MDA) breast carcinoma cells. MSCs are often the primary targets of the microenvironment transformation in cancer [23]. Our previous proteomic analysis has revealed a high expression level of the tetraspan membrane protein CD9 and the presence of importin β1, programmed cell death-interacting protein 6 Alix and Ca^2+^-dependent phospholipid-binding protein Annexin A2 among other molecules in FEMX-I cell-derived CD133^+^ EVs [24, 25]. Thus, we engineered cells to express CD9-green fluorescent protein (GFP), resulting in *in vivo* labeling of EVs, which allows monitoring of their intracellular transport upon internalization by host cells. Upon exposure to EVs, CD9-GFP are found not only in the nuclei of the recipient cells, but also in nuclear envelope invagination-associated late endosomes (N-ALE), which constitute an intermediate structure for the delivery of EV-derived biomaterials into cell nuclei.

## RESULTS

### Generation and labeling of EVs

**Figure 1:**
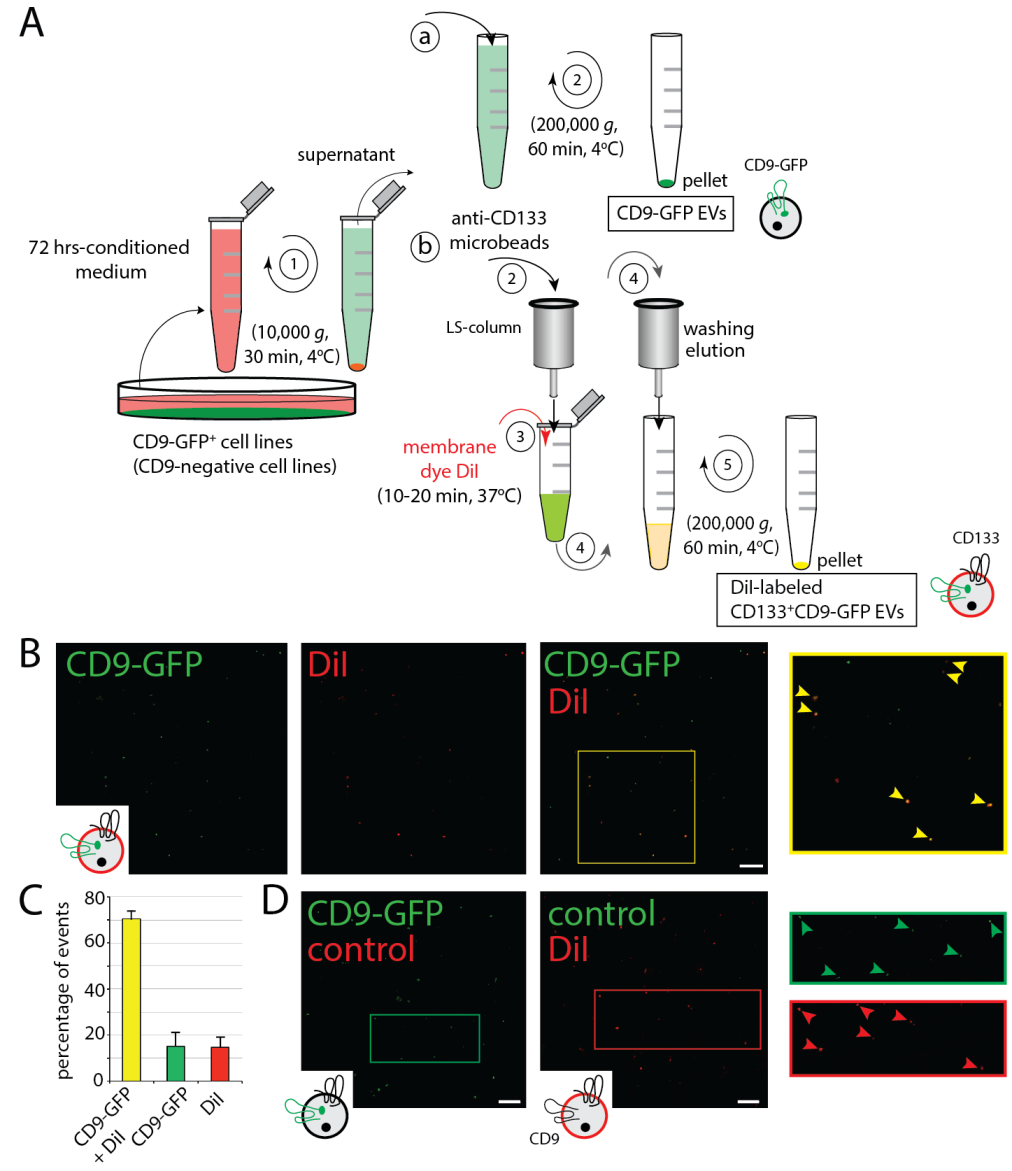
Production, Isolation and Labeling of EVs **A**. Isolation scheme of CD9-GFP^+^, CD133^+^CD9-GFP^+^ and CD133^+^CD9^-^ EVs derived from transfected or infected cells. EVs containing CD9-GFP (or those derived from cells infected with shCD9 lentivirus, not depicted) were enriched from 72 h-conditioned media by differential centrifugation alone (a) or in combination with immunomagnetic isolation using anti-human CD133 microbeads (b). In both cases, conditioned media were first centrifuged at 10,000 x *g* for 30 min (step 1). Total CD9-GFP^+^ EVs were then recovered upon ultracentrifugation of 10,000 *g*-supernatant (a, 2) or CD133^+^CD9-GFP^+^ EVs were immune-isolated (b, 2), labeled with membrane dye DiI (3), washed/eluted (4) and recovered by ultracentrifugation (5). The illustrated EVs indicated their CD9-GFP (green) and/or membrane (red) labeling. **B**., **C**. FEMX-I cell-derived DiI-labeled CD133^+^CD9-GFP+ EVs were adhered to coverslip and observed by CLSM (B). The area indicated with a yellow square is displayed at higher magnification. Arrowheads indicate double fluorescent EVs. Percentages of double-labeled EVs (CD9-GFP + DiI) or single-labeled EVs (CD9-GFP or DiI) within our preparations are presented (C, *n* = 3 independent experiments). Note that not all CD9-GFP^+^ EVs are labeled with DiI or expressed CD9-GFP. **D**. As controls, CD9-GFP^+^ EVs (left panel) or native EVs labeled with DiI (right panel) were observed by CLSM. The areas indicated with squares are displayed at higher magnification. Note the absence of autofluorescence of EVs in both conditions (arrowheads). Scale bar, 5 µm.

To trace the intracellular trafficking of EVs upon internalization by recipient cells, we engineered malignant FEMX-I and MDA tumor cells and primary MSCs to express CD9-GFP fusion protein, resulting in the production of *in vivo*-labeled EVs. CD9-GFP^+^ EVs released in the conditioned culture medium were enriched by differential centrifugation (Figure 1Aa). The average size of EVs produced by FEMX-I, MDA and MSCs was 123 ± 3.77 nm (mean ± standard error of the mean (s.e.m.)), 134.8 ± 1.00 nm and 114.6 ± 0.83 nm, respectively (Supplementary Figure 1A, *n* = 5 independent preparations). Their heterogeneity in terms of size was previously observed by electron microscopy [24]. In addition to CD9-GFP fluorescence, we stained FEMX-I cell-derived CD9-GFP^+^ EVs with membrane dye 1,1’-dioctadecyl-3,3,3’,3’-tetramethylindocarbocyanine perchlorate (DiI) upon their immunoisolation using CD133-paramagnetic beads (Figure 1Ab). About 70% of CD133^+^CD9-GFP^+^ EVs were positive for DiI as observed by confocal laser-scanning microscopy (CLSM) using the fluorescein isothiocyanate (FITC) and tetramethylrhodamine (TRITC) channels, respectively (Figure 1B, 1C). No red or green autofluorescence associated with EV was observed when DiI labeling was omitted or native CD133^+^ EVs were stained with it, respectively (Figure 1D, left and right panels, respectively).

### EV-derived macromolecular membrane complexes are transported into the nuclear compartment

**Figure 2:**
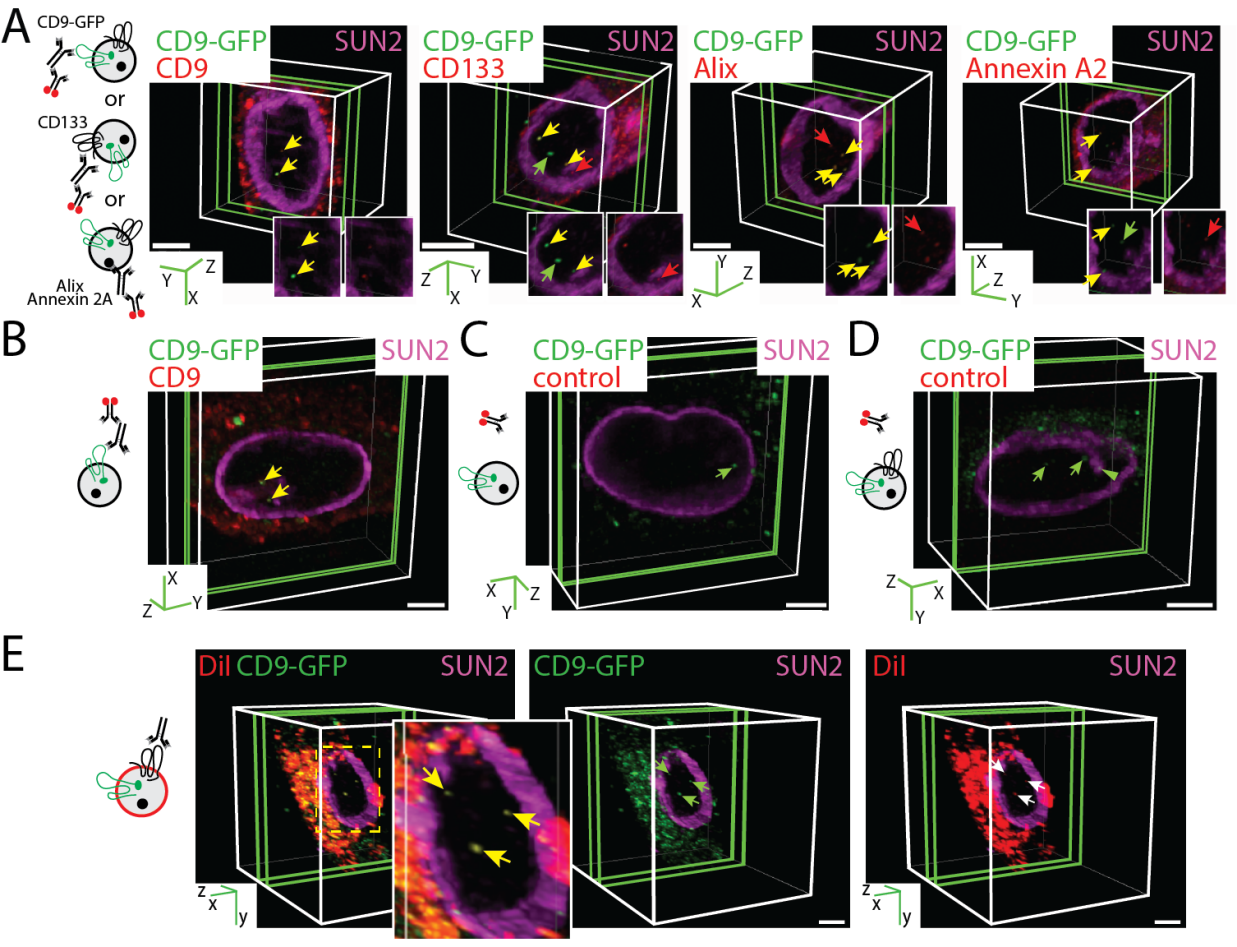
CD9-GFP ^+^ EV-derived Biomaterials Localized in the Nuclear Compartment of Recipient Cells **A**., **B**. FEMX-I cells (A) and MSCs (B) were incubated (4.5 h) with 5 × 10^7^ EVs/ml derived from CD9-GFP-transfected FEMX-I or infected MSCs, respectively, prior to their immunolabeling with SUN2 Ab (purple, all cases) and CD9, CD133, Alix and Annexin A2 Abs (red) as indicated. Cells were analyzed by CLSM and 3D reconstructions of 1-3 sections (0.4-µm each for FEMX-I cells or 0.2-µm for MSCs, green slice) of one cell are shown. In insets, relevant areas of the nucleus are displayed in individual single channels. (A). CD9-GFP or specific antigen signals and those co-labeled are indicated (green, red and yellow arrows, respectively). The cartoons illustrate the EVs with the pertinent labeling. Note the absence of CD133 in MSC-derived EVs. **C**., **D**. As negative control, only secondary Ab was employed on MSCs (C) and FEMX-I cells (D). The green arrowhead indicates the presence of CD9-GFP in nuclear envelope invagination (D). **E**. FEMX-I cells were incubated with DiI-labeled CD133^+^CD9-GFP^+^ EVs (5 × 10^7^ particles/ml) prior to CLSM. Arrow indicates the double-labeled EV-derived biomaterials in the nucleus. Scale bars, 5 µm.

To determine whether isolated EV-derived biomaterials reach the nuclear compartment of recipient cells as previously suggested (see Introduction), we incubated native FEMX-I, MDA and MSCs with enriched CD9-GFP^+^ EVs (5 × 10^7^ particles/ml; 0.075 µg protein/ml) for 4.5 h. In each case, EVs were generated by the corresponding CD9-GFP-transfected or infected cell lines. After the incubation, inner nuclear membrane was stained with SUN domain-containing protein 2 (SUN2) antibody (Ab) and samples were analyzed by CLSM. Data are presented as three-dimensional (3D) image of one cell where a slice composed of 1-3 sections (0.4 µm/section for FEMX-I and MDA cells, 0.2 µm/section for MSCs) containing the relevant biomaterials in the nuclear compartment is shown. In all cases, GFP signals were detected in the nucleus of receiving cells (Figure 2A, FEMX-I cells; 2B, MSCs; MDA, data not shown). They appeared nonetheless with a low frequency per cell, i.e. 1.87 ± 0.03, 1.96 ± 0.05 and 1.44 ± 0.02 [50 cells were evaluated per experiment, *n* = 3 independent experiments] for FEMX-I, MDA and MSCs, respectively.

To gain insight into the composition of nuclear EV-derived biomaterials, we probed FEMX-I cells or MSCs with specific Abs directed against EV-associated proteins. Indirect immunofluorescence revealed first that a significant fraction of nuclear GFP materials was co-stained with CD9 Ab in both cell types (Figure 2A, first panel; 2B). Indeed, more than half of GFP were immunopositive for CD9, suggesting that CD9-GFP fusion protein is not fully degraded (for quantification see Supplementary Figure 1B). Second, the stem (and cancer stem) cell marker CD133 - a pentaspan membrane glycoprotein - was also associated with nuclear GFP in FEMX-I cells (Figure 2A, second panel, Supplementary Figure 1B). CD133 is not expressed in cultured MSCs or MDA cells [26] (see below). Third, Alix and Annexin A2 were also co-stained with nuclear GFP (Figure 2A, two right panels, respectively). Alix is a *bona fide* protein involved in exosome biogenesis [27] and it is found, like annexin 2A, in FEMX-I cell-derived CD133^+^ EVs [25]. In all cases, a fraction of nuclear GFP was not labeled with any Ab, suggesting that they represent a proteolytic fragment of CD9-GFP (Figure 2A, Supplementary Figure 1B). Single antigens were also observed, suggesting that they are either degradation products of EVs or derived from host cells (Figure 2A, Supplementary Figure 1B). No antigenic fluorescent signal was detected when primary Ab was omitted (Figure 2C, 2D). In contrast, the non-EV associated multidrug resistance protein 1, expressed in both FEMX-I cells and MSCs, was not co-detected with nuclear GFP (data not shown).

The association of specific proteins with nuclear GFP signal suggests that a macromolecular complex derived from EVs is transported to recipient cell nuclei. This finding prompted us to determine whether the multiprotein complex remains associated with the lipid bilayer of EVs. To that end, we stained the CD133^+^CD9-GFP^+^ EVs with DiI prior to their incubation with FEMX-I cells. CLSM analysis revealed double-fluorescent (DiI and GFP) signals in nuclei of host cells, suggesting that not only a multiprotein complex was transferred to the nuclear compartment, but also the associated DiI-labeled membrane fragments (Figure 2E).

### EV-derived biomaterials are found in nuclear envelope invagination-associated late endosomes

**Figure 3:**
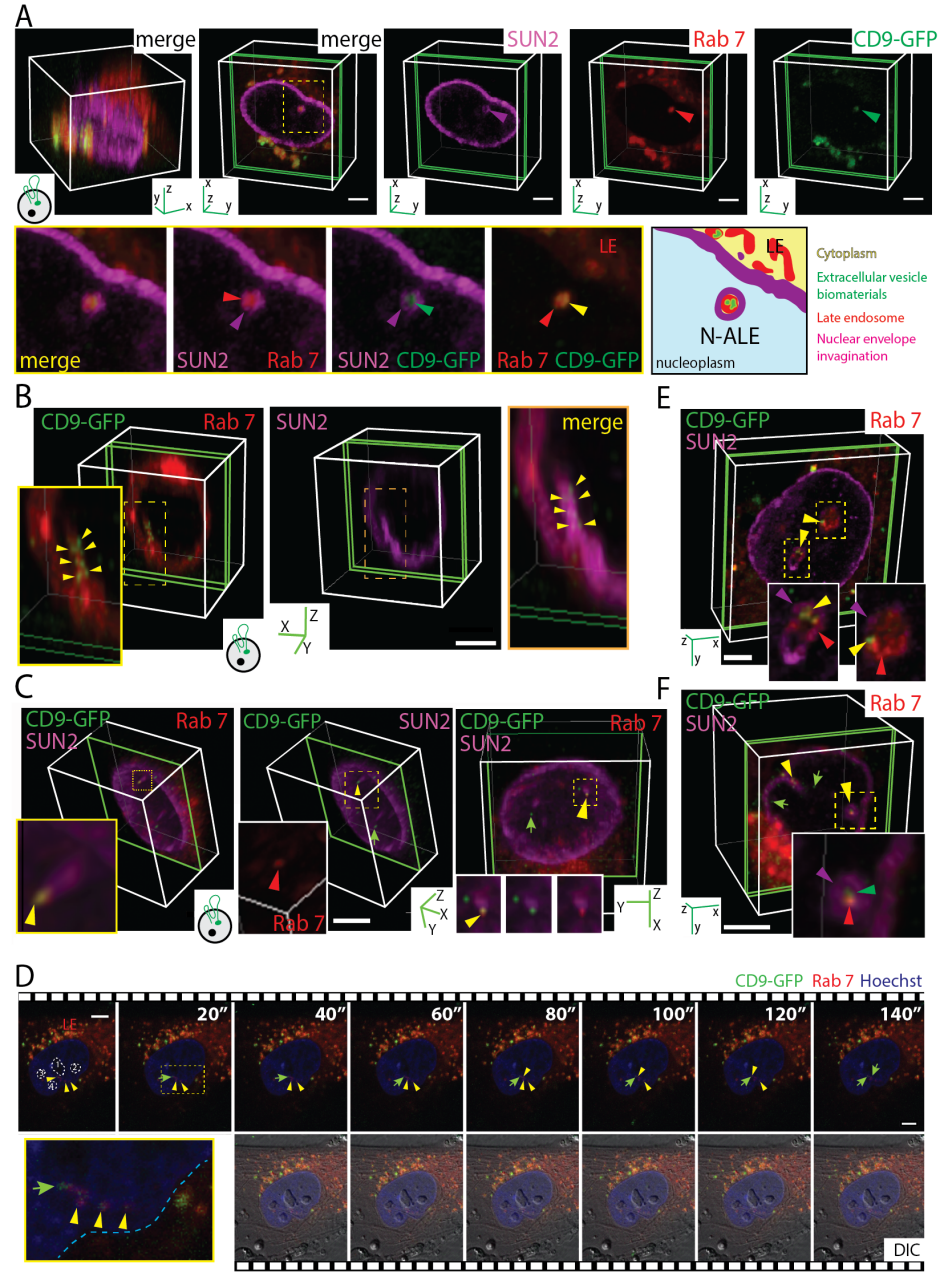
CD9-GFP ^+^ EV-derived Biomaterials Localized in N-ALE **A**.-**F**. MSCs (A, D, E) or FEMX-I cells (B, C, F) expressing Rab7-RFP (A-D) or not (E, F) were incubated (4.5 h) with 5 × 10^7^ CD9-GFP^+^ EVs/ml derived from CD9-GFP^+^ MSCs or FEMX-I cells, respectively, prior to staining with SUN2 Ab (A-C) or Hoechst 33342 (D). Alternatively, cells were double-labeled with SUN2 and Rab7 Abs (E, F). Samples were analyzed either by CLSM (A-C, E, F) or time-lapse video microscopy (D). 3D reconstructions of 40 x-y sections (0.4-µm each; A, left panel) or 1-3 sections (green slice) of a given cell are shown (A-C, E, F). Elapsed time of video is indicated in top right corner (D). Relevant areas (dashed squares) covering nuclear envelope invaginations containing Rab7^+^ late endosomes (LE) and CD9-GFP (purple, red and yellow/green arrowheads, respectively) are enlarged. A cartoon illustrating N-ALE containing CD9-GFP is displayed (A). Note the presence of CD9-GFP at the tip of the N-ALE (C. yellow arrowhead) or in the nucleoplasm (C, D, green arrow). N-ALE are often close to the nucleoli (D, numbered 1-4). DIC, differential interference contrast image. Scale bars, 5 µm. Images in panels C and F are presented in Supplementary Videos 1 and 2, respectively.

To investigate the intracellular path followed by EV-mediated extracellular signals *en route* to the nuclear compartment, we infected stromal and cancer cells with baculovirus that produced red fluorescent protein (RFP) or GFP-tagged fusion proteins that highlight specific cellular organelles, e.g., late and early endosomes, Golgi apparatus, ER and mitochondria. For instance, late endosomes were identified using Rab7-RFP. Afterward, MSCs and FEMX-I cells were incubated (4.5 h) with CD9-GFP^+^ EVs (5 × 10^7^ particles/ml) that derived from the same cell type as the recipient ones, processed for immunolabeling for SUN2 and analyzed by CLSM. Surprisingly, CD9-GFP was not only scattered throughout the nuclear compartment, but also concentrated in SUN2-labeled nuclear envelope invaginations (Figure 3A, MSCs; 3B, 3C, FEMX-I cells; data not shown; see also Figure 2D). Therein, the late endosomal membranes highlighted by Rab7-RFP appeared surrounded by the inner nuclear membrane and contained CD9-GFP (Figure 3A-3C), indicating that a subdomain of late endosomes is penetrating into nuclear envelope invaginations. Thereafter, these particular late endosome subdomains are referred to as N-ALE. Transverse and longitudinal sections of N-ALE containing CD9-GFP are presented (Figure 3A and 3B, 3C, respectively). In some cases, Rab7 and CD9-GFP are found at the extremity of nuclear envelope invagination (Figure 3C, Supplementary Video 1). We observed a limited number of CD9-GFP-loaded N-ALE per cell (i.e. 0.275 ± 0.06 [51 MSCs cells analyzed], 0.26 ± 0.05 [107 FEMX-I cells] and 0.39 ± 0.07 [90 MDA cells]), which is in line with the low number of nuclear CD9-GFP signals.

The occurrence of EV-derived CD9-GFP in N-ALE was also detected in live cells by time-lapse video microscopy (Figure 3D), indicating that they are not false signals generated during the fixation. Furthermore, the CD9-GFP appeared to be transferred to the nucleoplasm (Figure 3D, arrow) suggesting that N-ALE may act as an intermediate subcellular compartment in the transmission of biomaterials between the extracellular milieu and nuclei of host cells.

Since the overexpression of Rab proteins (including Rab7) can induce the neo-formation of biological structures or interfere with endosomal functions [28, 29], we immunolabeled the endogenous Rab7 instead of its ectopic expression as a fusion protein. Again, CD9-GFP is found in Rab7+ late endosomes located in the nuclear envelope invaginations (Figure 3E, MSCs; 3F, Supplementary Video 2, FEMX-I cells), indicating that Rab7-RFP overexpression did not cause this phenomenon.

### Characterization of N-ALE

**Figure 4:**
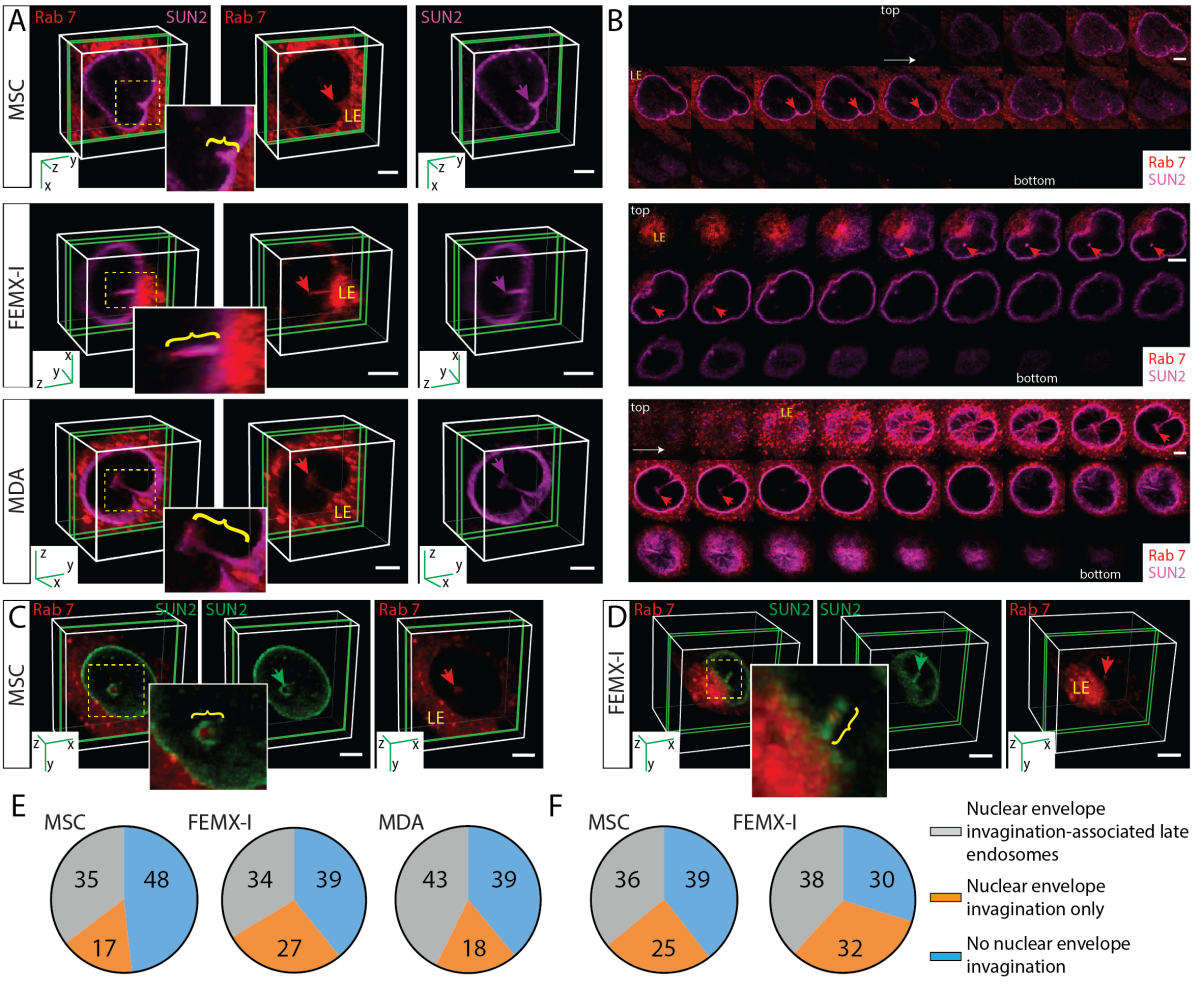
Subdomains of Late Endosomes Invading Nuclear Envelope Invaginations **A**., **B**. MSCs, FEMX-I and MDA cells expressing Rab7-RFP were immunolabeled with SUN2 Ab and analyzed by CLSM. 3D reconstructions of 1-3 sections (green slice) of one cell are shown, and relevant areas (yellow squares) covering nuclear envelope invaginations (purple arrow) with Rab7^+^ late endosomes (red arrow) are enlarged (A). The late endosome subdomains invading nuclear envelope invagination are indicated (bracket). Relevant individual x-y sections of the images presented in panel A are displayed (B). The image of FEMX-I cells is also presented in Supplementary Video 3. **C**., **D**. MSCs or FEMX-I cells were double-labeled with SUN2 and Rab7 Abs and analyzed by CLSM. The late endosome subdomains (Rab7, red arrow) invading nuclear envelope invagination (SUN2, green arrow) are indicated (bracket). **E**., **F**. Percentages of cells with SUN2-stained nuclear envelope invaginations containing Rab7 (observed either upon its expression as Rab7-RFP fusion protein (E) or immunostaining (F)), or not are indicated in pie charts (>50 cells were evaluated per experiment, *n* = 3; extended data are presented in Supplementary Table 1). Scale bars, 5 µm.

Does the incubation of cells with EVs induce N-ALE? To answer this question, we re-analyzed each of the three cell types (MSCs, FEMX-I and MDA cells) without exposure to CD9-GFP^+^ EVs. In all cell types, N-ALE were observed, irrespective of the methods of detection of Rab7 (i.e. ectopic expression of Rab7-RFP *versus* immunostaining) (Figure 4A-4D). Nonetheless, not all cells contained N-ALE. They were observed in ≈ 35-45% of them (Figure 4E, 4F, Supplementary Table 1). In contrast to late endosomes, early endosomes and Golgi apparatus that were highlighted with Rab5a- or Golgi-resident enzyme N-acetylgalactosaminyltransferase 2-RFP fusion proteins, respectively, were not observed in the nuclear envelope invaginations (Supplementary Figure 2A-C). Given the membrane continuity between ER and the outer nuclear membrane, a co-localization of ER luminal marker (i.e. GFP containing ER signal sequence of calreticulin with KDEL retention signal) with Rab7 and SUN2 was detected in N-ALE (Supplementary Figure 2D). Mitochondria highlighted with GFP-fusion protein containing the leader sequence of E1 α pyruvate dehydrogenase were excluded (Supplementary Figure 2E).

At a molecular level, the nuclear-intermediate filament protein lamin B1 that constitutes the lattice-like matrix at the inner membrane of nuclear envelope was found in the nucleoplasm surrounding the N-ALE (Supplementary Figure 3A). β-actin appeared to be excluded or its amount therein was below the detection level (Supplementary Figure 3B). Structurally, N-ALE were observed either as superficial indentations of nuclear envelope (Figure 4A, MSCs) or deep recesses (Figure 3A, MSCs; Figure 4A, Supplementary Video 3, FEMX-I). Often, they came in close contact with nucleoli (Figure 3D). Multiple and/or branching N-ALE were detected (Figure 4A, MDA; Supplementary Figure 4A, B, FEMX-I) consistent with the biology of nuclear envelope invaginations [21].

### EVs can modulate the dynamics of N-ALE

**Figure 5:**
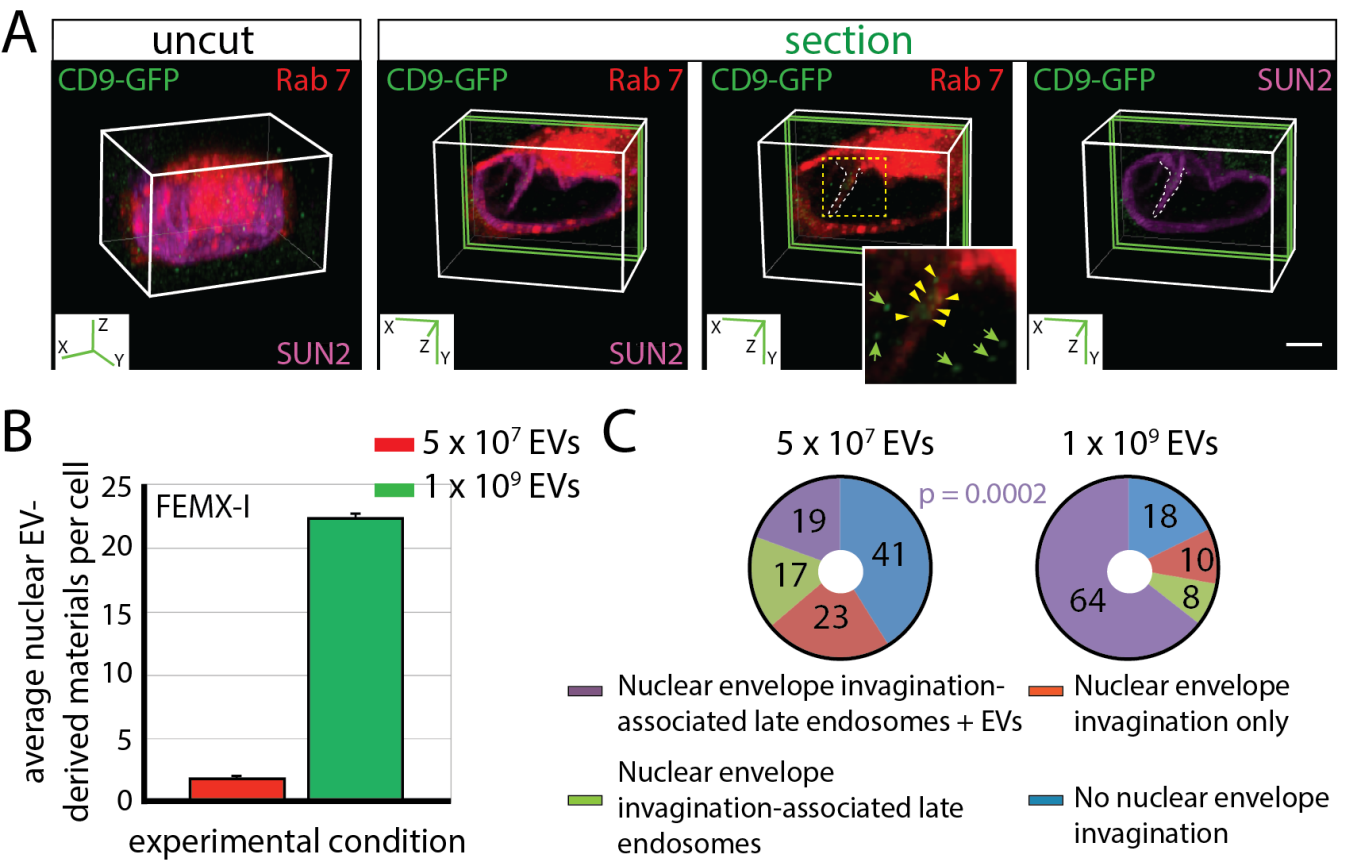
Dose-dependent Localization of CD9-GFP^+^ EV-derived Biomaterials in N-ALE and Nuclear Compartment of Recipient Cells **A**.-**C**. FEMX-I cells expressing Rab7-RFP were incubated (4.5 h) with 5 × 10^7^ or 1 × 10^9^ EVs/ml derived from CD9-GFP-transfected FEMX-I cells prior their immunostaining for SUN2, and analyzed by CLSM. 3D reconstructions of 40 x-y sections (0.4-µm each; uncut) or 2 sections (green slice) of cells incubated with 1 × 10^9^ EVs/ml are displayed. The relevant area (dashed square) covering a nuclear envelope invagination (dashed line) with Rab7^+^ late endosomes containing CD9-GFP (arrowheads) is enlarged. CD9-GFP found in the nucleus are indicated (arrows). The average of CD9-GFP in the nucleus of a given cell is indicated (B, error bars, s.e.m., >50 cells were evaluated per experiment, *n* = 3). Percentages of cells without or with SUN2-stained nuclear envelope invaginations containing or not Rab7-RFP (late endosomes) as well as CD9-GFP (EV-derived biomaterials) upon their exposure to EVs are indicated in pie charts (C, extended data are presented in Supplementary Table 1). The P value of the number of cells with N-ALE containing CD9-GFP (N-ALE + EVs) upon exposure to 5 × 10^7^ *vs*. 1 × 10^9^ CD9-GFP^+^ EVs/ml is indicated. Scale bar, 5 µm.

Although a limited amount of EV-derived biomaterials was observed in N-ALE and nuclei of recipient cells, we cannot exclude that a prolonged exposure to, or a higher concentration of, EVs will increase the number of N-ALE and/or modulate their frequency. Indeed, incubation of FEMX-I cells with CD9-GFP^+^ EVs for 18 h (instead of 4.5 h) increased the nuclear EV-derived biomaterials from 1.87 ± 0.03 up to 3.16 ± 0.18 (*p* = 0.002). The exposure of cells to a higher concentration of EVs (1 × 10^9^ instead 5 × 10^7^ particles/ml) resulted also in a significant increase in the number of CD9-GFP in the nuclear compartment (Figure 5A, 5B). Similarly, the percentage of cells with CD9-GFP-loaded N-ALE increased (Figure 5C, Supplementary Table 1), suggesting that EVs can modulate the dynamics of N-ALE and nuclear envelope invaginations [30]. Moreover, a 3.62-fold increase in CD9-GFP-loaded N-ALE per cell was observed (0.94 ± 0.08 *vs*. 0.26 ± 0.05; 50 cells were evaluated per experiment, *n* = 3), substantiating a link between the amount of EV-derived biomaterials in N-ALE and nuclei.

### Involvement of tetraspanin membrane protein CD9 in the endocytosis of EVs

Because tetraspanin protein CD9 is widely expressed in EVs, we evaluated its role in the uptake of EVs, and hence, the dynamics of N-ALE. To that end, we added a monoclonal (m) anti-CD9 Ab to recipient cells during their incubation with EVs (5 × 10^7^ particles/ml). First, we observed that the percentage of cells with CD9-GFP-loaded N-ALE significantly increased for FEMX-I and MDA cells, but marginally for MSCs (Supplementary Figure 5A, Supplementary Table 1). Second, the number of CD9-GFP-loaded N-ALE per cell almost doubled for cancer cells (from 0.26 ± 0.05 to 0.48 ± 0.06 [106 cells] and 0.39 ± 0.07 to 0.59 ± 0.07 [113 cells] for FEMX-I and MDA cells, respectively), but not for MSCs (0.275 ± 0.06 to 0.34 ± 0.07 [62 cells]). Third, the number of EV-derived biomaterials in the nucleus also increased; from 1.87 ± 0.03 to 3.2 ± 0.34 (Supplementary Figure 5B; *p* = 0.0177), 1.96 ± 0.05 to 2.92 ± 0.11 (*p* = 0.001), and 1.44 ± 0.02 to 1.94 ± 0.15 (*p* = 0.03) for FEMX-I, MDA and MSCs, respectively (50 cells evaluated per experiment, *n* = 3). These observations are in line with the role of N-ALE as an intermediate subcellular compartment in the transfer of EV-derived biomaterials to the nucleus of recipient cells. When a similar experiment was performed with an anti-CD133 mAb, no significant increase in the number of nuclear EV-derived biomaterials was detected in recipient cells (Supplementary Figure 5B). It is conceivable that CD9 Ab causes CD9 cross-linking, which stimulates the binding of CD9-containing EVs to the plasma membrane of host cells and enhances their uptake. Given the fusogenic role of CD9 [31, 32], the CD9 Ab may as well facilitate membrane fusion between EVs and recipient cells, particularly in N-ALE. The high level of CD9 in EVs derived from FEMX-I and MDA cells (Supplementary Figure 5C) by comparison to those produced by MSCs might explain the limited effect of CD9 Ab on MSCs (Supplementary Figure 5A).

To determine further the implication of CD9 in the uptake of EVs and the nuclear translocation of biomaterials derived therefrom, we knocked down its expression in FEMX-I and MDA cells using a non-overlapping pool of shRNAs (Supplementary Figure 5C, Cells). As a result, a significant reduction of CD9 was observed in EVs (Supplementary Figure 5C, top panels, EVs). The reduction of CD9 did not affect the presence of CD133 and importin β1 in EVs (Supplementary Figure 5C, middle and bottom panels, EVs). MDA cells did not express CD133 (data not shown). DiI-labeled CD133^+^CD9-depleted EVs were then prepared from FEMX-I CD9sh cells and incubated with either native or CD9-depleted FEMX-I cells (Supplementary Figure 5D, see cartoon). While DiI-labeled membrane signals from native EVs used as positive control were found in the nuclear compartment of native cells (Supplementary Figure 5D, top panels, arrow), the endocytosis of CD9-depleted EVs was strongly decreased (Supplementary Figure 5D, second row panels). Reciprocally, the cellular uptake of CD9-containing EVs by CD9-depleted cells was reduced (Supplementary Figure 5D, third row panels). Almost no cytoplasmic EV-derived biomaterials were observed when EVs and host cells were depleted of CD9 (Supplementary Figure 5D, bottom panels). In all scenarios, the depletion of CD9 either in EVs or recipient cells impaired the delivery of EV-derived biomaterials to N-ALE and completely abolished their nuclear entry (Supplementary Figure 5D). The latter phenomenon was also observed when CD9 Ab was added during the incubation (Supplementary Figure 5D). Similar data were obtained with MDA CD9sh cells and DiI-labeled CD9-depleted EVs (data not shown). Given the absence of CD133 in MDA cells, EVs were enriched from 10,000 *g*-supernatant of conditioned medium by ultracentrifugation.

Beyond the role of CD9 in the uptake of EVs, the general perturbation of endocytic machinery interferes with nuclear entry of EV-derived biomaterials. For instance, the pre-treatment of cells with dynasore, a cell-permeable inhibitor of dynamin, totally blocked the intracellular uptake of DiI-labeled CD133^+^CD9-GFP^+^ EVs (Supplementary Figure 6A), and consequently the nuclear localization of EV-derived biomaterials. Likewise, inhibition of cholesterol-dependent endocytic process by methyl-β-cyclodextrin-mediated cholesterol extraction partly impeded EV uptake, but totally abrogated the nuclear localization of EV-derived biomaterials (Supplementary Figure 6A). Finally, isolated MSC nuclei were not able to uptake the content of EVs (Supplementary Figure 6B), indicating that CD9-mediated endocytosis is a prerequisite condition for the nuclear localization of EV-derived biomaterials.

### Implication of importin β1 in the nuclear entry of EV-derived biomaterials

**Figure 6:**
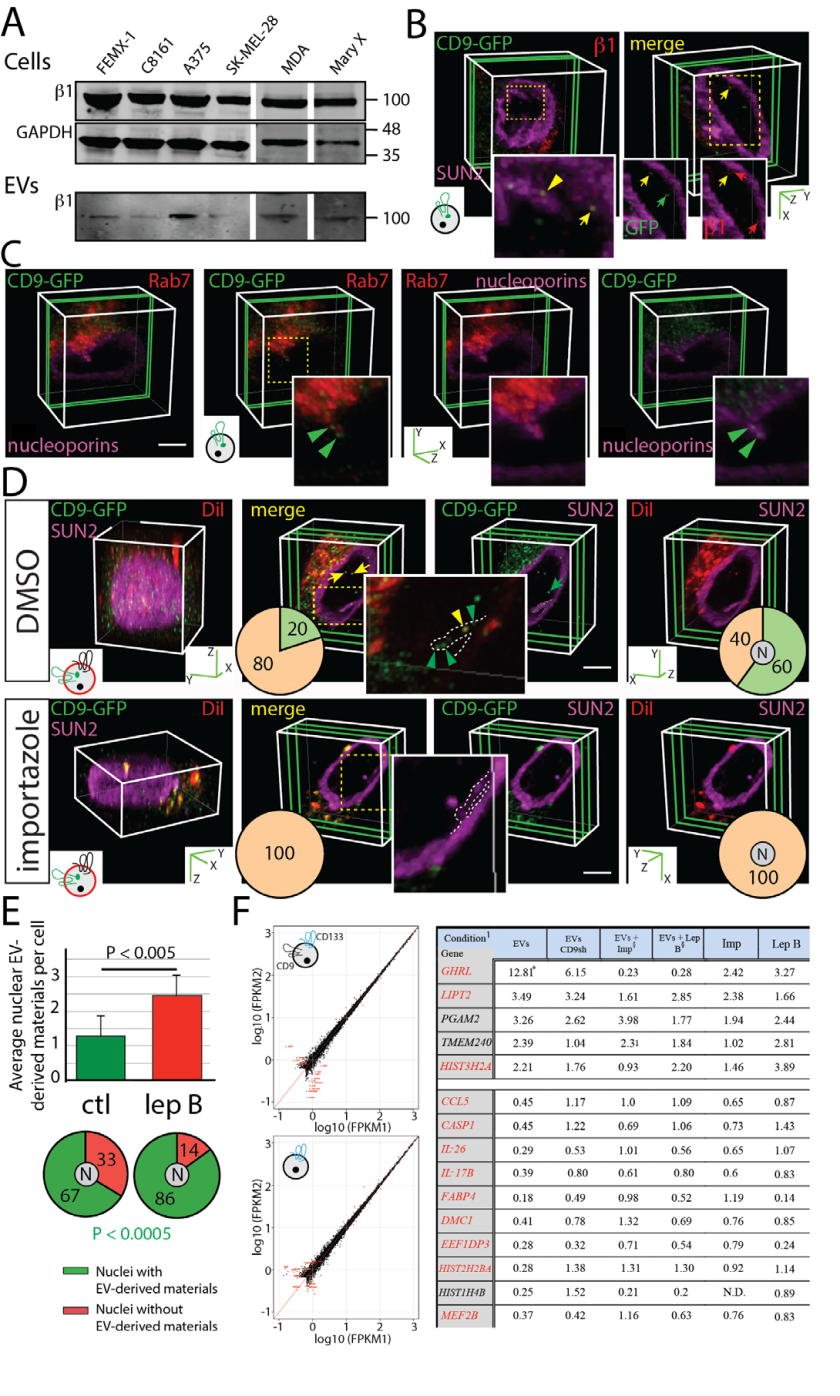
Nuclear Pore Involvement in the Translocation of EV-derived Biomaterials into the Nucleus and Early Changes in MSC Transcriptome Induced by Melanoma EVs are Prevented by Importazole **A**. Lysates from melanoma and breast cancer cell lines and EVs released therefrom were analyzed by immunoblotting for importin β1. GAPDH is used as loading control. (For the blot source data, see Supplementary Figure 9). **B**., **C**. Native FEMX-I cells (B) or those expressing Rab7-RFP (C) were incubated (4.5 h) with FEMX-I cell-derived CD9-GFP^+^ EVs (5 × 10^7^ EVs/ml, see cartoon). Cells were then double-immunolabeled for SUN2 and importin β1 (B) or nucleoporin alone (C), and analyzed by CLSM. Representative images of 3D reconstructions of 1-3 sections (green slice) are displayed. Relevant areas (dashed squares) are enlarged. A partial colocalization of CD9-GFP and importin β1 in N-ALE and nucleus are indicated (B, yellow arrowhead and arrow, respectively) (B). Both proteins are also individually detected in the nucleus (B, green and red arrow). A colocalization CD9-GFP and nucleoporins in Rab7^+^ structures is found (C, green arrowhead). **D**., **E**. FEMX-I cells were incubated (4.5 h) with DiI-labeled CD133^+^CD9-GFP^+^ EVs (5 × 10^7^ EVs/ml). Importazole (D) and leptomycin B (E) were added after the initial 2.5 h-incubation. Dimethyl sulfoxide (DMSO) (D) and methanol (E) were used as vehicle controls (ctl). Cells were immunolabeled for SUN2 and analyzed by CLSM. 3D reconstructions of 40 to 45 x-y sections (0.4-µm each; D, left panels) or 3 sections (D, right panels, green slice) of one cell are shown. The presence of CD9-GFP and DiI-labeled membranes in SUN2-labeled nuclear envelope invagination (dashed line) or nucleus are indicated (D, arrowhead and arrow, respectively). Percentages of cells with EV-derived biomaterials in nuclear envelope invagination or nucleus (N) are indicated in pie charts (D, left and right, respectively; positive, green; negative, orange) while the average of nuclear EV-derived biomaterials per cell and percentages of cells with nuclear EV-derived biomaterials are presented (E) The P value of the number of cells with nuclear EV-derived biomaterials is indicated (>50 cells were evaluated per experiment, *n* = 3; extended data are presented in Supplementary Table 1). Scale bars, 5 µm. **F**. Scatter plot of log_10_ Fpkm (Fragments Per Kilobase Of Exon Per Million Fragments Mapped) of MSC genes exposed to solvent alone (1) or to 1 × 10^9^ EVs/ml from parental (upper left panel) or CD9sh (lower left panel) FEMX-I cells for 4.5 h (2). Fold-changes in gene expression compared with control MSCs exposed to solvent alone (right panel). ^1^Conditions are described in Supplemental Methods. *All numbers indicate the fold change in gene expression by comparison to the control sample (i.e. MSCs exposed to solvent alone). ^§^Imp, importazole; Lep B, leptomycin B.

The immunoblotting of cell lysates and EVs derived from various human melanoma and breast cancer cell lines revealed a widespread expression of importin β1 in EVs (Figure 6A) in agreement with our proteomic analysis of FEMX-I cell-derived CD133^+^ EVs [25]. Importin β1 mediates nuclear transportation of cytoplasmic proteins through the nuclear pore complex. This stepwise process is regulated by the interaction of importin β1/importin α/cargo complex with nucleoporins [33–35]. Although importins are known to translocate soluble cargo into the nucleus, an additional role to shuttle EV-derived biomaterials from N-ALE to nuclei cannot be excluded. Indeed, the low number of CD9-GFP-loaded N-ALE per cell and limited amount of CD9-GFP signal in the nucleus might be explained by the fact that only a minute fraction of total nuclear pores (i.e. those in close contact with late endosomal membranes) is involved in the nuclear import of EV-derived biomaterials. These observations prompted us to examine the potential co-localization of importin β1 with endocytosed EVs. Interestingly, importin β1 immunoreactivity co-localized with EV-derived CD9-GFP inside the N-ALE and nuclei of recipient FEMX-I cells (Figure 6B, yellow arrowhead and arrow, respectively). Likewise, nucleoporin immunoreactivity is found in nuclear membrane invaginations containing Rab7 and EV-derived CD9-GFP (Figure 6C, green arrowhead).

The treatment of host cells with importazole, a molecule that specifically inhibits the function of importin β1 by altering its interaction with Ran-GTP [36], totally abrogated the nuclear localization of fluorescent materials derived from DiI-labeled CD133^+^CD9-GFP^+^ EVs (Figure 6D, bottom panels). In contrast, 60% of cells treated with DMSO as a vehicle control contained nuclear DiI/CD9-GFP signals (Figure 6D, top panels, yellow arrow). Intriguingly, we found no EV-derived biomaterials in nuclear membrane invaginations of importazole-treated cells, while 20% of DMSO-treated cells contained them, suggesting that their maintenance therein is dependent on the nuclear import system (Figure 6D). The exposure of cells to importazole neither interfered with the presence of Rab7^+^ late endosome subdomains in nuclear envelope invaginations (38 and 34% of importazole- and DMSO-treated cells, respectively, contain N-ALE, 30 cells per experiments, *n* = 3) nor impaired the trafficking of EVs to cytoplasmic-located Rab7^+^ late endosomes (data not shown). Conversely, we observed increases in the number of nuclear DiI/CD9-GFP signals per cell and the percentage of cells with such nuclear labeling (Figure 6E, top and bottom panels, respectively, Supplementary Table 1) when recipient cells were incubated with leptomycin B, which impairs the nuclear export of cargo molecules. Collectively, these data suggest a role of the nuclear translocation machinery in transfer of EV-derived biomaterials into the nucleus, and therein they may have a short life.

### Changes in MSC transcriptome induced by nuclear import of EV-derived biomaterials

Are EV-derived biomaterials translocated to nuclear compartment influencing the transcriptome of recipient cells? By applying a RNA sequencing (RNA-seq) approach, we found that the expression of only 15 transcripts was altered upon short exposure (4.5 h) of MSCs to EVs derived from cancer FEMX-I cells, but not (or to a lesser extent) to CD9-depleted EVs (Figure 6F). *Ghrelin (GHRL), LITP2, PGAM2, TMEM240* and *HIST3H2A* were up regulated, whereas *CCL5, interleukin-26 (IL-26), interleukin-17B (IL-17B), Casp1, FABP4, DMC1, HIST2H2BA, HIST1H4B*, *EEF1DP3* and *MEF2B* were down regulated. Interestingly, the changes in expression level of 11 of the 15 genes were prevented after the addition of either importazole or leptomycin B, suggesting that their regulation relied on the nuclear translocation of EV-derived biomaterials (Figure 6F, red). Five identified gene products (ghrelin, IL-26, IL-17B, Casp1 and CCL5) are known to be involved in inflammatory processes.

To validate the transcriptome, we evaluated by real-time quantitative reverse-transcription PCR (qRT-PCR) the upregulation of three selected MSC-associated genes upon exposure of MSCs to FEMX-I cell-derived EVs in the absence or presence of importazole. The qRT-PCR confirmed the increase of GHLR and LIPT2 expression upon incubation of EVs, by comparison to control samples (i.e. without the addition of EVs), in an importazole dependent manner (Supplementary Figure 7A, 7B). Interestingly, PGAM2 displayed also a significantly increase (Supplementary Figure 7A, B), although the impact of importazole was less striking (Supplementary Figure 7A, 7B) in line with the transcriptome (Figure 6F). GAPDH transcript expression was not affected under these conditions (data not shown).

### Occurrence of N-ALE in breast cancer patient biopsies

**Figure 7:**
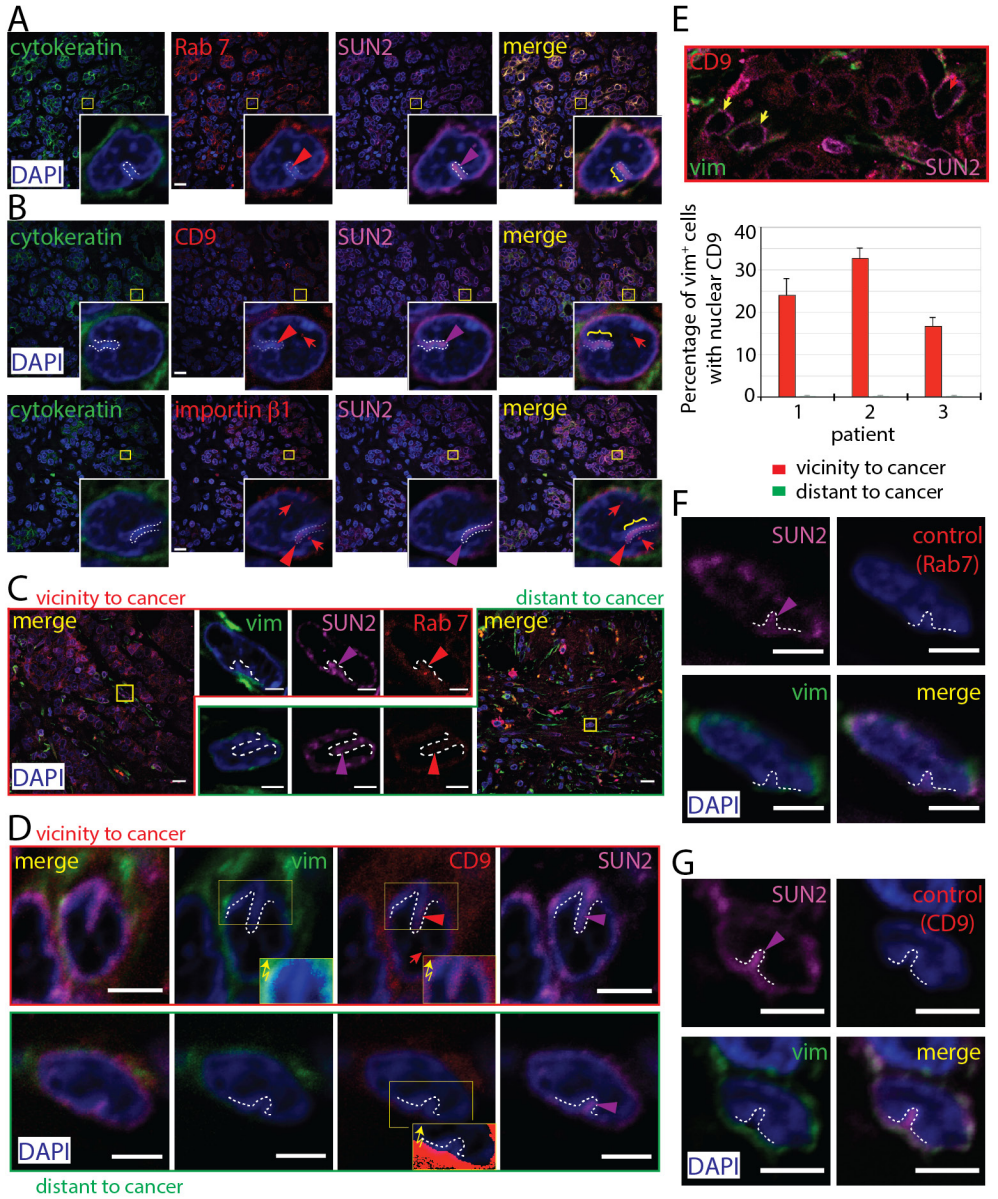
Presence of N-ALE in Breast Cancer Patient Biopsies **A**.-**E**. Biopsies from patients 1 (A, B, E), 2 (E), and 3 (C, D, E) were immunolabeled either for pan-cytokeratin (CK) or vimentin (vim) in combination with Rab7, CD9, importin β1 or SUN2 followed by the appropriate secondary Abs. Samples were counterstained with 4’,6-diamidino-2-phenylindole (DAPI) and analyzed by CLSM. An overview (0.25-µm section) of tissues is presented and relevant areas (squares) covering N-ALE (brackets and/or dashed lines) with Rab7 (A, C), CD9 or importin β1 (B, D) signals are enlarged. To detect a potential weak staining of vim or CD9 in N-ALE, the non-modified exposure of the immunofluorescence was manually increased using Photoshop software (D, insets with zigzag arrows). Note the absence of CK and vim in N-ALE as well as CD9 in those found in stromal cells distant from tumor microenvironment (TME). Quantification of CD9 in vim^+^ cell nuclei (E). The nuclear CD9 immunoreactivities in vim^+^ cells in vicinity or distant regions of TME were evaluated by analyzing 41-45 x-y sections (0.25-µm each) covering the entire nucleus (>100 cells from three patients were evaluated). Yellow arrow shows vim^+^ cell without CD9 in their nucleus. **F**., **G**. As negative controls, primary Ab against Rab7 (F) or CD9 (G) was omitted. In all panels, colored arrowhead indicates the presence of the corresponding immunofluorescent signal in N-ALE, while red arrow shows the nuclear CD9 staining. Scale bars, 15 (overview panels, A-C), 3 (C, D, F, G) µm.

We then expanded our *in vitro* observations to human carcinomas *in situ*. Breast carcinoma biopsies were immunolabeled for cytokeratin (CK) and vimentin (vim) to distinguish breast carcinoma cells from stromal cells, respectively. First, we observed the presence of Rab7 immunoreactivity in invaginations of the nuclear envelope of CK^+^ cancer cells, indicating that N-ALE are detected in tissue biopsies (Figure 7A). Second, both CD9 and importin β1 were found in N-ALE and nuclei of cancer cells (Figure 7B). Although we cannot exclude that both proteins originated from intracellular recycling pools, the latter data are consistent with EV origin. Third, we observed Rab7^+^ labeling in nuclear envelope invaginations of a fraction of vim^+^ stromal cells in the immediate tumor microenvironment (TME) and in those more distant (>2 mm) from the carcinoma cells (Figure 7C). In those found in immediate TME, the N-ALE demonstrated CD9 immunoreactivity, which was not the case in distant areas (Figure 7D). Both CK and vim were excluded from N-ALE (Figure 7A-7D). Interestingly, 15-35% of cancer-associated stromal cells had nuclear CD9 signals at a frequency of 1.36 ± 0.14 (s.e.m.) per nucleus, while this event was rarely observed in distant stroma (Figure 7E, >100 vim^+^ cells were evaluated per patient, *n* = 3). The difference could be attributed to the relative quiescence of stromal cells outside the TME, as opposed to the highly proliferating cancer-associated stromal cells [37]. A decrease in signal-mediated nuclear transport between proliferating and quiescent cells, due to changes in nuclear pores, was reported [38] as well as alterations in the structure of nuclear pores during quiescence in plant cells [39].

### Lack of EV-derived biomaterials in nuclei of quiescent MSCs

**Figure 8:**
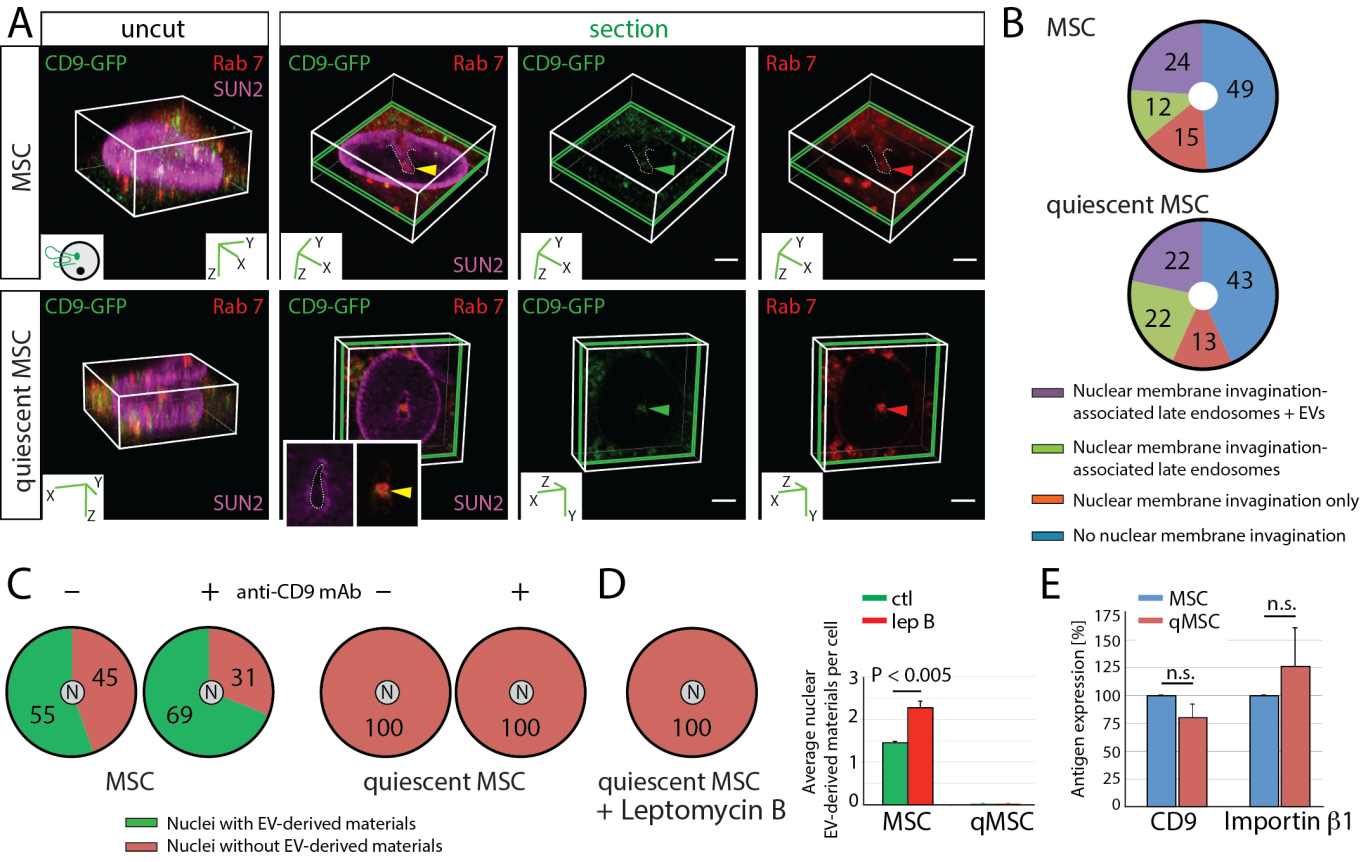
Lack of EV-derived Biomaterials in Nuclei of Quiescent MSCs **A**.-**D**. Proliferating and quiescent MSCs expressing Rab7-RFP were incubated (4.5 h) with FEMX-I-derived CD9-GFP^+^ EVs (5 × 10^7^ particles/ml) in the absence (A-C) or presence (C) of anti-CD9 mAb (25 µg/ml) prior the immunostaining for SUN2. Cells were analyzed by CLSM. Alternatively, leptomycin B (lep B, 10 ng/ml) was added after the initial 2.5 h-incubation with EVs (D). Arrowheads indicate CD9-GFP (green) and Rab7 (red) in nuclear envelope invagination (dashed lines, yellow) (A). Percentages of cells without or with SUN2-labeled nuclear envelope invagination containing or not Rab7-RFP (late endosomes) and CD9-GFP (+ EV) are indicated in pie charts (B, *n* = 3, extended data are presented in Supplementary Table 1). Percentages of cells with or without CD9-GFP in their nucleus (N) are indicated (C, D, extended data are presented in Supplementary Table 1) while bar graph shows the average number of nuclear CD9-GFP per cell in the presence or absence of leptomycin B (D). **E**. The expression of CD9 and importin β1 in proliferating (MSC) and quiescent (qMSC) MSCs were quantified by immunoblotting and normalized to GAPDH as internal loading control. Antigen expression was arbitrarily set at 100% for proliferating MSCs. N.s., not significant. Scale bars, 5 µm.

To evaluate whether cell quiescence can influence the transport of biomaterials from cancer cell-derived EVs to the nucleus of host cells, we conducted *in vitro* experiments with proliferating *versus* quiescent MSCs (Figure 8). Their cell cycle was evaluated by flow cytometry using propidium iodide staining (Supplementary Figure 8). We used CD9-GFP^+^ EVs produced by FEMX-I melanoma cells. As observed with proliferating MSCs, Rab7^+^ late endosomes penetrating the nuclear envelope invaginations were detected in quiescent MSCs in agreement with stromal cells in more distant areas from the TME (Figure 8A). The percentage of quiescent MSCs with CD9-GFP-loaded N-ALE was not different from proliferating MSCs (Figure 8B). Thus, the endocytosis of EVs and the sorting of EV-derived biomaterials to N-ALE are not impaired in quiescent MSCs. However, we found no quiescent MSCs with CD9-GFP in their nucleus even in the presence of anti-CD9 Ab during cell-EV incubation (Figure 8C). The lack of EV-derived biomaterials in quiescent MSC nuclei is neither explained by an activated export of cargo proteins, as shown by the addition of leptomycin B (Figure 8D), nor by a reduction of CD9 or importin β1 (Figure 8E). At first glance, the dissolution of nuclear envelope during mitosis may be the critical issue for the nuclear entrapment of EV-derived biomaterials. Since the doubling time of MSCs is much greater than 24 h [40], and solely ≈6% of proliferating cells are in G2 + M phases (Supplementary Figure 8), these observations suggest that for the majority of proliferating MSCs (i.e. 55%) that have CD9-GFP in their nucleus, undergoing mitosis is not required for nuclear accumulation of EV-derived biomaterials. All together, these data suggest that changes in nuclear pores might be one of the causes for the lack of nuclear EV-derived biomaterials in quiescent stromal cells.

## DISCUSSION

Here, we present evidence that subdomains of the Rab7^+^ late endosomes come together with nuclear envelope invaginations to form a new sub-nuclear compartment where biomaterials from EVs are delivered. Therein, EV-derived biomaterials are transferred to the nucleus where they modify the gene expression profile of the host cells. In cancer cell niches, this trafficking mechanism could be particularly important because of the growing evidence that cancer cells hijack the surrounding tissue microenvironment to promote their own growth and spreading [7].

The discovery of N-ALE as distinctive endosomal subdomains is consistent with recent reports of distinct signaling pathways engaged by c-Met depending on different (peripheral/perinuclear) endosomal subdomains [41]. Upon internalization, several receptors remain actively involved in signaling [42], indicating that endosomes are not simply a desensitization mechanism [43]. The intracellular pathway used by EV-derived biomaterials *en route* to the nucleus is also in line with studies showing new endosomal pathways that deliver surface proteins to the nucleus [44, 45]. Such mechanism might explain the presence of EV-associated proteins and nucleic acids in nuclei of target cells [17–19], and the atypical nuclear localization of CD9 and CD133 in cancer cells [46, 47].

Throughout this study, we employed 5 × 10^7^ or 1 × 10^9^ particles/ml of EVs, which are in the range of EV concentrations found in the plasma of cancer patients [10]. The amount of EVs might be a limiting factor for the N-ALE-mediated nuclear translocation, particularly if the EV diffusion is regulated by a source-sink mechanism [48]. The latter process could explain why cancer cell-derived EVs are taken up by stromal cells within the immediate TME, but not by those in distant areas. Specific biological conditions of recipient cells might also regulate the functionality and dynamics of N-ALE. For instance, modifications in nuclear pore complexes, as observed between quiescent and proliferating cells [38], may explain the lack of EV-derived biomaterials in the nuclei of quiescent MSCs. Being composed of two different cell membrane structures (i.e. nuclear envelope membrane invagination and a late endosome subdomain), N-ALE can be regulated by several mechanisms involving intrinsic and extrinsic factors. Thus, intrinsic factors influencing the formation and/or stabilization of nuclear envelope invaginations, e.g., nuclear intermediate filaments, can control the organization of N-ALE (reviewed in Ref. [21]). Extracellular factors (soluble or membrane-bound) and regulators of endocytosis may also modulate the dynamics of N-ALE. As an example, we showed that the exposure of cells to EVs increase the number of those containing EV-loaded N-ALE which is concomitant to a reduction of cells without nuclear envelope invaginations.

Functionally, the transcriptome of MSCs exposed to cancer cell-derived EVs indicates that nuclear import of EV-derived biomaterials is required for some of the early changes in gene expression. To support the survival and progression of tumors, cancer cell-derived EVs contribute to the neutralization of the anticancer immune response, participating in all known mechanisms by which cancer evades the immune system, such as differentiation and activation of immune suppressor cells, modulation of antigen presentation, and induction of T cell apoptosis. The five EV-dependent regulated genes whose expression changes were prevented by importazole (i.e. *ghrelin, IL-26, IL-17B, Casp1* and *CCL5)* may be relevant for the pro-tumorigenic activity of cancer EVs. The melanoma EV-induced up-regulation of ghrelin, a 28-residue peptide hormone that accelerates the growth of MSCs and has an anti-inflammatory activity [49], is consistent with the down-regulation of IL-26, IL-17B, caspase-1 and CCL5. In fact, IL-26 and IL-17B up-regulate expression of several pro-inflammatory cytokine genes [50, 51]; caspase-1 is an inflammatory response initiator [52]; and CCL5 plays an active role in recruiting leukocytes into inflammatory sites and in inducing the proliferation and activation of certain natural-killer cells [53]. Collectively, the cancer cell-derived EVs may predispose the contents of the cellular niche for cancer development and/or formation of metastases by shutting down the molecular players involved in inflammation responses. Of course, the physiological implication of N-ALE might be far beyond cancer-related events, as illustrated here, and cell biological phenomena such as proliferation, differentiation and cell migration can be regulated, at least in part, by this intermediate subcellular compartment.

**Figure 9:**
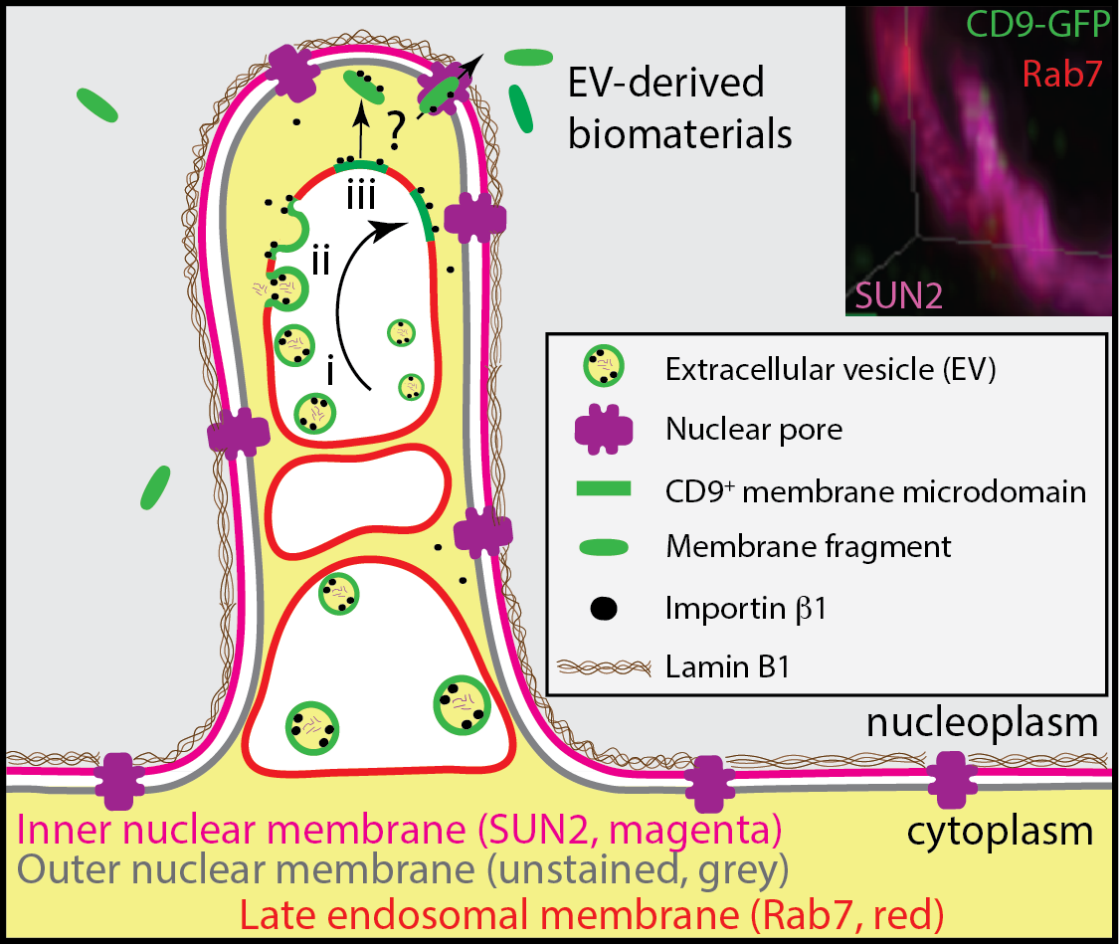
Model of N-ALE Endocytosed CD9-GFP^+^ EVs containing importin β1 are concentrated in N-ALE. Therein, they may fuse with endosomal membranes (steps i-iii), and hence release and concentrate their soluble content within the space created between endosomal and nuclear membranes, whereas their membrane components may translocate into nucleoplasm *via* nuclear pores. How EV-derived protein-lipid complexes stay intact upon fusion with endosome membrane and translocate to nucleoplasm through nuclear pores, which are size restricted, remain to be identified (question mark). A role of CD9^+^ membrane microdomains can be postulated.

Despite our findings, open questions remain to be investigated: (i) How can EV-derived membrane fragments pass through the nuclear pores that are size restricted (≈40 nm), and enter the nucleus? (ii) How are fragments of lipid bilayer of the EV-endosome membranes extracted when cargo proteins/importin β1 travel through the nuclear pore (Figure 9, question mark)? (iii) Are the CD9/CD133-containing membrane micro/nano-domains involved? (iv) Are importins involved in the retention (and/or extraction) of EV-derived biomaterials in (or from) N-ALE, as suggested by the importazole experiment? It is tempting to speculate that specific tetraspanin-enriched membrane microdomains and/or ordered-phase lipid domains, such as lipid rafts, may act as specific membrane platforms in the retention and extraction processes. The enrichment of membrane raft-associated lipids in EVs [25, 54] and the dynamic interaction of Annexin A2 with endosomal membranes that possess raft-like characteristics might be significant in this context [55]. The recent discovery of 8-12 nm EVs allows to hypothesize that very small EVs are transported or extracted from endosomal membranes as a whole through the nuclear pores [56].

Additional studies are required to dissect all potential avenues generated by the discovery of N-ALE. For example, a certain activity of miRNAs can occur in N-ALE as a subcellular compartment. The fusion of EVs with endosomal membranes will result in an accumulation of miRNA and other nucleic acids carried by EVs in the cytoplasm located between the endosomal membrane and the outer nuclear membrane. Therein, they might interact with their targets coming out of the nucleus. Alternatively, mature miRNAs can be translocated from cytoplasm to nucleus, as recently reported. Although the site of RNA-induced silencing complex has traditionally been considered the rough ER [57, 58], recent studies suggested that miRNAs are also transported into the nucleus, where they modulate their own biogenesis and that of other miRNAs [59], being involved in transcriptional and post-transcriptional regulatory processes at the nuclear level [60].

In conclusion, the N-ALE-mediated transfer of EV-derived biomaterials from cancer cells to the nuclear compartment of other cells (normal or cancerous) within the cancer niche may be essential for both autocrine and paracrine effects of cancer EVs; interference with the N-ALE pathway may be exploited for the development of innovative anti-cancer strategies. In physiological conditions, exchange of biomaterials between cells *via* N-ALE might modulate the properties of resident stem cells within their niche. The latter issue can be relevant for the development of clinical applications for tissue/organ injury. Since this novel subdomain of late endosomes penetrating into nuclear envelope invaginations often appears as a sword in its scabbard, we propose to call N-ALE “spathasomes” (from the Greek/Latin words “*spathi*/*spatha*” for sword).

## MATERIALS AND METHODS

Additional methods can be found in the Supplemental Information.

### Cell culture

The human MDA breast carcinoma cell line was obtained from the American Type Culture Collection. The FEMX-I cell line was originally derived from a lymph node metastasis of a patient with malignant melanoma [61]. FEMX-I cells were highly metastatic in immunodeficient mice [61, 62] and were found to be wild type for BRAF (A. Lorico, unpublished observation), PTEN and NRAS (E. Hovig, personal communication). Both cell lines were cultured in RPMI-1640 (Mediatech) containing 10% fetal bovine serum, 2 mM L-glutamine, 100 U/mL penicillin and 100 μg/mL streptomycin. Cells were used between passages 3 and 15. Cell lines were authenticated by morphology, proteomics and gene expression analysis as described [63].

Human bone marrow-derived MSCs, isolated from bone marrow aspirates from normal adult donors after informed consent as described [64], were obtained from Dr. D. J. Prockop (Texas A&M) and prepared under a protocol approved by the Texas A&M Institutional Review Board. MSCs were used between passages 2 and 5. Their multipotency was monitored by differentiation into adipocytes and osteoblasts as described [65]. To obtain MSCs in a quiescent state, cells were plated at 20,000 per 35-mm dish and grown to confluence for seven days.

### Enrichment of EVs

EVs were enriched from 72 h-conditioned media by differential centrifugation alone or in combination with immunomagnetic isolation using anti-human CD133 microbeads (Miltenyi Biotec) as described previously [25]. For the differential centrifugation, conditioned medium was centrifuged at 10,000 x *g* for 30 min at 4°C, and the resulting supernatant centrifuged at 200,000 x *g* for 60 min at 4°C. The pellet was resuspended in 200 µl PBS. For the isolation of CD133^+^ EVs, the anti-human CD133 Ab conjugated to paramagnetic microbeads was added to the 10,000 *g*-supernatant, and immunolabeled CD133^+^ EVs were enriched using LS-column (Miltenyi Biotec). Each of five independent EV preparations from each cell line or primary MSCs was examined by nanoparticle tracking analysis (NTA) for size distribution. Details are provided in Supplemental Methods.

### Incubation of cells with EVs

CD9-GFP^+^ EVs or DiI-labeled CD133^+^CD9-GFP^+^ EVs were added (5 × 10^7^ particles/ml, i.e. 0.075 µg protein/ml) to 1 × 10^5^ recipient cells for 4.5 h at 37°C. Higher concentration of EVs (1 × 10^9^ particles/ml, 1.5 µg protein/ml) was also used as indicated in figure legends. In some experiments, DiI-labeled CD133^+^ EVs were derived from FEMX-I shCD9 cells. When necessary, cells were infected with baculovirus for 24-48 h to express markers of early or late endosomes, ER, Golgi apparatus or mitochondria prior the addition of EVs (see above). For CD9 functional assay, after 3 h of incubation with EVs, 25 µg/ml CD9 Ab (Supplementary Table 2) was added to cells for another 90 min. When EVs were incubated directly with nuclei, the latter were prepared using NE-PER Nuclear and Cytoplasmic Extraction reagents (Thermo Scientific). Briefly, cells were trypsinized, washed with PBS, resuspended in ice-cold cytoplasmic extraction reagent (CER) I solution, vortexed and incubated on ice for 10 min. Ice-cold CER II was then added and cells were further incubated on ice for 1 min, vortexed and centrifuged at 16,000 x *g* for 5 min. The supernatant was removed and nuclei resuspended in culture medium, incubated with DiI-labeled FEMX-I-derived EVs (5 × 10^7^ particles/ml) for 4.5 h at 37°C and stained with 5 μg/ml Hoechst 33342 (Molecular Probes) for the last 30 min of incubation. As control, the nuclei of intact MSCs exposed to EVs were processed as well prior to imaging.

### CLSM

Cells in cultures and tissue samples were analyzed by CLSM using a Nikon A1R+ inverted confocal microscope with a 60X Apo-TIRF oil-immersion objective and a numerical aperture of 1.49 at either 512 × 512 or 1024 × 1024 pixel resolution. 405, 488, 561, and 638 nm solid-state lasers were used to excite DAPI/Hoechst 33342, GFP/FITC, TRITC/RFP/DiI and Cy5/Alexafluor 640, respectively, and corresponding fluorescence emissions were collected using 425-475, 500-550, 570-620 and 662-737 nm longpass filters. Details are provided in Supplemental Methods.

### Statistical analysis

All *in vitro* experiments were performed at least in triplicate. A minimum of 50 cells was analyzed in each experiment, if not indicated. Error bars in graphical data represent means ± s.e.m. Unless indicated, statistical analysis was determined using a two-tailed Student's t-test, and p-values inferior to 0.05 were considered significant. Statistical details are presented in Supplementary Table 1.

### Accession to RNA sequencing data

The raw data of RNA sequencing reported in this paper are accessible since October 1, 2016. Reviewers may use the following link to access the data http://www.ncbi.nlm.nih.gov/geo/query/acc.cgi?token=epslweikbxabpcr&acc=GSE83559.

## SUPPLEMENTARY MATERIALS FIGURES, TABLES AND VIDEOS









## Abbreviations

CK: cytokeratin
CLSM: confocal laser-scanning microscopy
DiI: 1,1’-dioctadecyl-3,3,3’,3’-tetramethylindocarbocyanine perchlorate
EVs: extracellular membrane vesicles
FITC: fluorescein isothiocyanate
GFP: green fluorescent protein
MDA: MDA-MB-231
MSCs: multipotent mesenchymal stromal cells
N-ALE: nuclear envelope invagination-associated late endosomes
RFP: red fluorescent protein
TME: tumor microenvironment
TRITC: tetramethylrhodamine
vim: vimentin
