# Supplementary material for: Nuclear transport of cancer extracellular vesicle-derived biomaterials through nuclear envelope invagination-associated late endosomes
Source: Oncotarget. 2017 Jan 24;8(9):14443–61. doi: 10.18632/oncotarget.14804 (PMC5362417; doi:10.18632/oncotarget.14804)
Supplement: Supplementary file 1 [file oncotarget-08-14443-s001.pdf]

Nuclear transport of cancer extracellular vesicle-derived biomaterials through nuclear envelope invagination-associated late endosomes

Supplemental Methods

Chemicals

Dynasore (80 µM; Santa Cruz Biotechnology) and importazole (40 µM; Sigma Aldrich) were dissolved in dimethyl sulfoxide (DMSO). The final DMSO concentration never exceeded 0.25%, and it was used as vehicle control when necessary. The stock solution of leptomycin B (5 µg/ml; Sigma Aldrich) in 70% methanol (v/v) was diluted in PBS to the final concentration of 10 ng/ml. Methyl-β-cyclodextrin (10 mM; Sigma Aldrich) was dissolved in water before use.

Antibodies

The primary antibodies (Abs) used for immunodetection and functional assay are listed in Supplementary Table 2. Secondary Abs utilized were tetramethylrhodamine (TRITC)-conjugated anti-mouse IgG (715-025-150, Jackson ImmunoResearch) or anti-rabbit IgG (711-025-152), Cy5-conjugated anti-rabbit IgG (711-175-152) or anti-mouse IgG (715-025-150) and fluorescein isothiocyanate (FITC)-conjugated anti-mouse IgG (GTX26669, GeneTex) or anti-rabbit IgG (GTX27050). For immunoblotting, IRDye 680RD anti-mouse IgG (926-68070, LI-COR Biosciences) was used.

Cell culture

For EV preparation, 6-well plates were pre-coated with 20 mg/ml poly(2-hydroxyethyl methacrylate) (poly-(HEMA); Sigma) in 95% ethanol and cells cultured in Dulbecco's modified Eagle's medium (Gibco) additioned with 2% B27 supplement (Life Tech), 2 mM L-glutamine, 100 U/mL penicillin and 100 µg/mL streptomycin.

The human A375 and SK-MEL28 melanoma cell lines were obtained from the American Type Culture Collection; the human C8161 melanoma cell line was obtained from G. Pizzorno; the human Mary-X inflammatory breast carcinoma cell line was obtained from S. H. Barsky. All cell lines were cultured in RPMI-1640 (Mediatech) containing 10% fetal bovine serum, 2 mM L-glutamine, 100 U/mL penicillin and 100 µg/mL streptomycin.

Transfection

FEMX-I and MDA cells were transfected with 10 μg PS100010 PrecisionShuttle mammalian plasmid encoding CD9 with a green fluorescent protein (GFP) tag at its C-terminus under the control of the cytomegalovirus promoter (RG202000; Origene) using FuGene (Promega) in a 1:3 DNA:lipid ratio. Cells were selected by introducing 400 μg/ml of G418 (Life Technology) in the culture medium for seven days, resulting in >99% GFP-positive cells. Antibiotics were removed from the medium at least one week before experiments.

Baculoviral and lentiviral vectors and infection

The baculovirus-based CellLight BacMam 2.0 Golgi-RFP, Early Endosomes-RFP and Late Endosomes-RFP encoding for Golgi-resident enzyme N-acetylgalactosaminyltransferase 2–, Rab5a– and Rab7–RFP fusion protein, respectively, were purchased from Life Technologies. Likewise for the ER-GFP and mitochondria-GFP encoding GFP fused to the ER signal sequence of calreticulin with KDEL retention signal or leader sequence of E1 α pyruvate dehydrogenase, respectively.

To express CD9-GFP fusion protein in MSCs, lentiviral particles containing the pCT-CD9-GFP plasmid (CYTO122-VA-1; System Biosciences) were utilized. The virus were preloaded onto retronectin-coated plates and centrifuged at 960 x *g* for 30 min at 4°C. The operation was repeated twice. The supernatant was then removed and plates were washed with PBS before addition of cells. After transduction, stable cell lines were selected by introducing 1 μg/ml puromycin in the culture medium for a week. Cells were kept in culture for a maximum of 5 passages (~3 weeks).

To inhibit CD9 expression, CD9 shRNA lentiviral particles (sc-35032-V, Santa Cruz Biotechnology) containing the puromycin resistant gene were employed [1]. They consist of a pool of concentrated, transduction-ready viral particles containing 3 target-specific constructs that encode 19-25 nt (plus hairpin) shRNA designed to knock down gene expression. Lentiviral particles containing shRNA construct encoding a scrambled sequence (sc-108080) were used as negative control. After transduction, stable cell lines were selected by introducing 2 μg/ml puromycin in the culture medium for a week and CD9-negative cells were sorted by flow cytometry.

Flow cytometry

FEMX-I and MDA CD9sh cells were incubated with HI9a anti-CD9 Ab for 60 min, washed, and incubated with Cy5-conjugated anti-mouse IgG secondary Ab for 30 min. CD9-negative

cells were isolated from transduced shCD9 cells using an SH800 cell sorter (Sony Global, Champaign, IL) and maintained in complete medium with puromycin (2 μg/ml). Antibiotic was removed from the medium at least one week before experiments.

Cell cycle analysis

Cell cycle of proliferating and quiescent MSCs was analyzed using the method of propidium iodide staining and analyzed on a SH800 cell sorter. Cell cycle distribution was calculated by FlowJo version 7 software.

Immunoblotting

Detergent cell lysate extracts obtained upon lysis of cells in a buffer (50 mM Tris-HCl pH 7.5, 100 mM NaCl and Set III protease inhibitor cocktail (Calbiochem)) containing 1% Triton X-100 were analyzed by sodium dodecyl sulfate–polyacrylamide-gel electrophoresis under reducing conditions except for CD9 and GAPDH when co-incubated with anti-CD9 Ab. 5,000 (MSC) or 30,000 (all other cell lines) cell equivalents were loaded per lane. After protein transfer, membranes were blocked with 1% bovine serum albumin in PBS for 16 h, and then probed with a given primary Ab (see Supplementary Table 2). After washing, membranes were incubated with an IRdye secondary Ab (LI-COR Biosciences) and visualized using an Odyssey CLx system (LI-COR Biosciences).

NTA

We used the light-scattering characteristics of 488-nm laser light on EV preparations undergoing Brownian motion injected by continuous flow into the sample chamber of a Nanosight LM10 unit (Malvern). Data are presented as the mean ± s.e.m of 5 independent preparations, where each is an average of six 30-sec video recordings.

Labeling of EVs with membrane dyes

$CD133^+$ EVs were labeled for 10-20 min at 37°C by the addition of 1,1-dioctadecyl-3,3,3,3-tetramethylindocarbocyanine perchlorate (DiI, Life Technologies) at a final concentration of 5 μM. Excess of dye was removed using LS-columns. DiI-labeled $CD133^+$ EVs were eluted with cold PBS, centrifuged at 200,000 x *g* for 60 min at 4°C and resuspended in 200 μl PBS. To exclude the possibility that magnetic beads alone formed clusters that nonspecifically

absorbed DiI, control CD133-coupled magnetic beads were treated with DiI prior to their incubation with recipient cells. Under these conditions no fluorescent dye was observed in cells (data not shown).

Drug treatments

Cell treatments with dynasore (80 μM) or methyl-β-cyclodextrin (10 mM) were initiated 30 min prior to addition of EVs for 4.5 h, while treatments with importazole (40 μM) or leptomycin B (10 ng/ml) were started after the initial 2.5 h of incubation with EVs and continued for the remaining 2 h. When necessary, the appropriate control vehicle (DMSO, methanol) was used. All experiments were repeated independently three times.

Immunocytochemistry

Cells treated under various condition and/or incubated with EVs were washed with PBS, fixed in 4% paraformaldehyde for 20 min at room temperature (RT), washed twice with PBS and permeabilized with 0.2% Tween 20 in PBS for 15 min at RT. Cells were then incubated with primary antibodies directed against SUN2, CD133, CD9, Alix, Annexin A2, Rab7, importin β1 or nucleoporins (see Supplementary Table 2) for 60 min at RT, washed twice with PBS, and incubated with appropriate fluorescent secondary antibodies for 30 min at RT. As a negative control, only secondary Ab was employed. The isolated MSC nuclei were stained with Hoechst 33342 (5 μg/ml) during the last 30 min of incubation with DiI-labeled EVs. Cells or isolated MSC nuclei were imaged in PBS by confocal laser-scanning microscopy (CLSM).

Immunohistochemistry

Formalin-fixed, paraffin-embedded patient biopsies (4-μm sections) of infiltrating ductal carcinoma of the breast were derived from anonymized archival sources within the Department of Pathology at UCLA. All Ab labeling, primary and secondary, were performed sequentially, except for pan-cytokeratin or vimentin that were detected simultaneously with CD9 or Rab7, overnight at 4°C with primary Abs that had been raised in different species. A sequential multiple immunofluorescence protocol which circumvented potential problems arising from using multiple primary Abs raised in the same host species (e.g., rabbit) was also employed [2]. As negative control, only secondary Ab was employed. Slides were then washed with PBS and stained with 1 μg/ml 4',6-diamidino-2-phenylindole (DAPI) for 15 min

at RT. Anti-fade mountant (ProLong Diamond, Thermo Fisher) was added to the slides, which were then mounted with 1.5-mm coverslips and viewed by CLSM.

CLSM and time-lapse video microscopy

All images were acquired under the same microscope settings for subsequent calculations of mean fluorescence and recorded using NIS Elements software (Nikon). Raw images were processed using Fiji [3]. The number of nuclear envelope invagination-associated late endosomes per cell and the localization of EV-derived biomaterials therein and/or nuclei from scanned samples were manually counted. Each z-section through the nucleoplasm (10-12 sections of 0.4 μm each for FEMX-I and MDA cells, 5-7 sections of 0.2 μm each for MSCs) was assessed individually and any observed positive fluorescent signal, e.g., GFP-tagged, DiI-labeled or antibody-stained EVs, were counted as EV-derived biomaterials and data collectively calculated. To determine the value of cytoplasmic DiI-staining (Supplementary Figure 5D), maximum intensity projections (MIPs) of z-stacks were made and thresholded to create ROI outlines of cells using the "analyze particles" function. Cell outlines were overlaid onto original z-stacks and DiI fluorescence was measured using Fiji and plotted as mean fluorescence per Z-slice.

For the live imaging, Rab7-RFP-expressing cells were incubated with CD9-GFP^{+} EVs (1 x 10^{9} particles/ml) at 37°C, 5% CO_2 humidified chamber for 4.5 h. Hoechst 33342 (5 μg/ml) was added for the last 60 min. Images were acquired at 20-second interval for 5 min with microscope described above. A short video was then created from the acquired time points using the NIS software.

RNA sequencing

To establish the early effect of FEMX-I-derived EVs on the transcriptome of MSCs in the absence or presence of modulators of nuclear uptake, differentially expressed genes in MSCs were analyzed by RNA-seq. 7 experimental groups in biological duplicates were analyzed, including solvent alone, EVs, EVs plus importazole, EVs plus leptomycin B, EVs from FEMX-I CD9sh, and importazole or leptomycin B alone. Samples with RNA Integrity Numbers of 8 or greater were prepped using the Illumina TruSeq mRNA Sample Prep v2 kit. mRNA prepared from total RNA (0.1-3 μg) was then fragmented and copied into first strand cDNA. The 3' ends of the cDNA were then adenylated and adapters ligated. The products were purified and enriched by PCR to create the final cDNA library. Libraries were checked

for quality and quantity, clustered on the cBot (Illumina) and sequenced on a HiSeq 2500 (Illumina) in High Output mode.

Bio-informatic analysis

To analyze RNA-seq data, NCBI human reference genome (GRCH38.84) and its GTF file and associated index files were downloaded from the Illumina iGenome website (http://support.illumina.com/sequencing/sequencing_software/igenome.html). TopHat2 software [4] was used to map short read sequences to the whole reference genome. The BAM alignment files were further processed by CuffDiff (version 2.2.1) for estimating transcripts' abundances and testing for differential expression between groups. Up- or down-regulated gene lists were selected using the cutoff criteria of log2 absolute fold change values > 1.1. RNA-Seq short read sequence files were submitted as DNA Sequencing Data (traces and short reads) to the NCBI Gene Expression Omnibus (GEO) database.

Real-time quantitative reverse-transcription PCR (qRT-PCR)

Total RNA collected from MSCs exposed for 4.5 h to solvent alone, FEMX-I-derived EVs, or EVs plus importazole were analyzed by two-step qRT-PCR. First, genomic DNA was removed in the RNA preparation using the DNase I recombinant, RNase-free kit (10U; Roche). Reaction was carried out at 37°C for 15 min followed by 10 min at 75°C to inactivate the enzyme. Afterward, SuperScript IV VILO master mix (Invitrogen), which includes both oligo (dt)18 and random hexamer primers, was added to reverse transcribe RNA. The samples were incubated 10 min at 25°C (primer annealing), 10 min at 50°C (reverse transcription) and 5 min at 85°C (enzyme inactivation). Second, PCR reaction plate was then setup using cDNA templates and the following reagents from Applied Biosystems: TaqMan gene expression master mix, TaqMan gene expression assays containing primers and specific 6-carboxyfluorescein reporter probes for GHRL (nucleotide sequence: 5'-AGAGGCCAAAGAGGCCCCAGCCGAC-3'; Assay ID, Hs01074053_m1), LIPT2 (5'-GCGCGATCGGAGTCCGCTGTGGAAG-3'; Hs00420102_g1), and PGAM2 5'-TGTCAAGCACCTGGAAGGGATGTCA-3'; Hs00165474_m1). GAPDH and 18S rRNA were utilized as housekeeping gene controls. Thermal cycling conditions were set using the following parameters: hold for 2 min at 50°C, hold for 10 min at 95°C, and 40 cycles of 15 sec at 95°C and 1 min at 60°C. Triplicate measurements for each gene of interest and their analysis were conducted using the 7500 Fast Real-Time PCR System (Applied Biosystems). The number of cycles required for the fluorescent signal to cross the threshold (C_T) was

determined by setting the baseline and threshold values between the 3rd and 15th cycle and the exponential growth phase of the amplification curve, respectively. To calculate ΔC_T and fold-changes, all quantitations were normalized to the GAPDH endogenous control.

Data availability

The raw data of RNA sequencing reported in this paper are accessible since October 1, 2016. Reviewers may use the following link to access the data http://www.ncbi.nlm.nih.gov/geo/query/acc.cgi?token=epslweikbxabpcr&acc=GSE83559

References

1. Rappa G, Green TM, Karbanova J, Corbeil D and Lorico A. Tetraspanin CD9 determines invasiveness and tumorigenicity of human breast cancer cells. Oncotarget. 2015; 6(10):7970-7991.

2. Lewis Carl SA, Gillete-Ferguson I and Ferguson DG. An indirect immunofluorescence procedure for staining the same cryosection with two mouse monoclonal primary antibodies. J Histochem Cytochem. 1993; 41(8):1273-1278.

3. Schindelin J, Arganda-Carreras I, Frise E, Kaynig V, Longair M, Pietzsch T, et al. Fiji: an open-source platform for biological-image analysis. Nat Methods. 2012; 9(7): 676-682.

4. Kim D, Pertea G, Trapnell C, Pimentel H, Kelley R and Salzberg SL. TopHat2: accurate alignment of transcriptomes in the presence of insertions, deletions and gene fusions. Genome Biol. 2013; 14(4):R36.

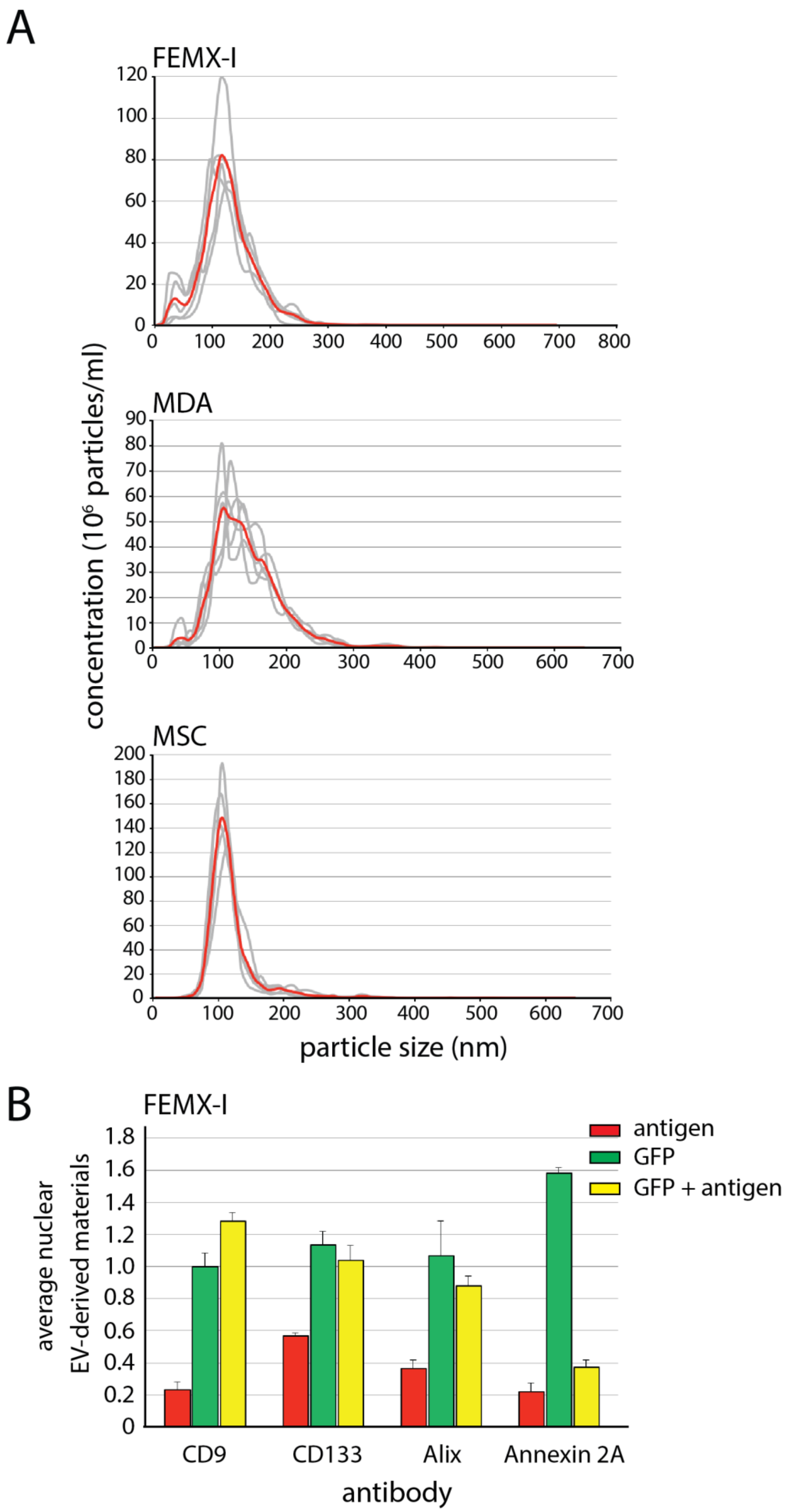
A
FEMX-I
MDA
MSC
concentration (10^6 particles/ml)
particle size (nm)
0
100
200
300
400
500
600
700
800
B
FEMX-I
antigen
GFP
GFP + antigen
average nuclear EV-derived materials
CD9
CD133
Alix
Annexin 2A
antibody

Supplementary Figure 1: Size of EVs Released by CD9-GFP$^+$ Cells and Quantification of EV-derived Macromolecular Complexes in the Recipient FEMX-I Cell Nucleus. A. CD9-GFP$^+$ EVs released from transfected FEMX-I and MDA cells or infected MSCs were analyzed by nanoparticle tracking. Average median size of particles is presented with a red line (n = 5). **B**. FEMX-I cells incubated (4.5 h) with FEMX-I cell-derived CD9-GFP$^+$ EVs were double-immunolabeled with Abs against SUN2 and CD9, CD133, Alix or Annexin A2. Nuclear materials containing either GFP alone (GFP, green) or in combination with a given antigen (GFP + antigen, yellow) or the antigen alone (antigen, red) were quantified (>50 cells were evaluated per experiment, n = 3). Data are derived from Figure 2A.

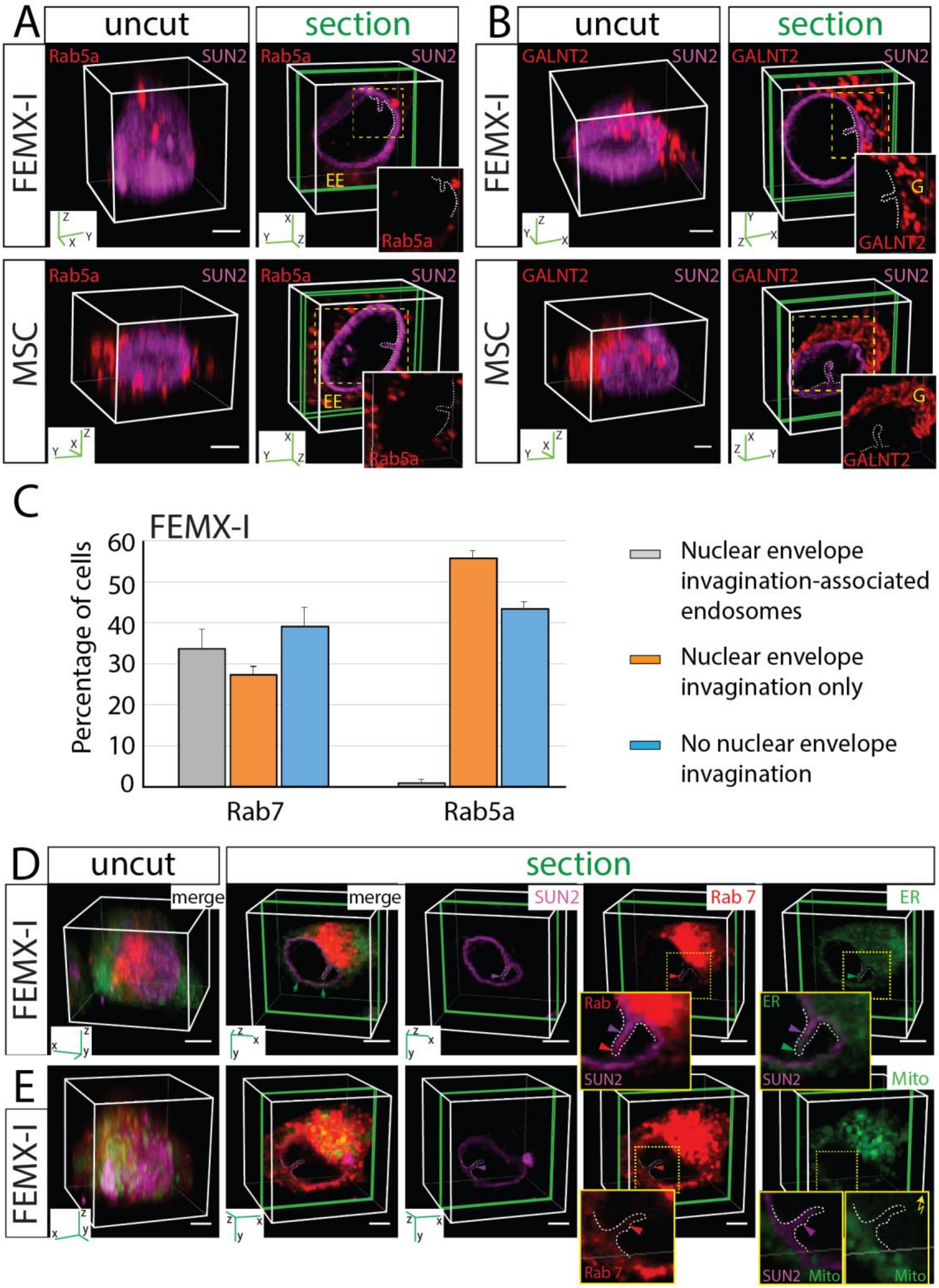

Supplementary Figure 2: Early Endosomes or Golgi Apparatus are Excluded from Nuclear Envelope Invaginations. A-C. FEMX-I and MSCs were infected with baculovirus encoding either Rab5a (A), GALNT2 (B) or Rab7 (not shown) –RFP fusion proteins that label early (EE) or late endosome, or Golgi apparatus (G), respectively, prior to their immunolabeling for SUN2 (purple). Samples were analyzed by CLSM. 3D reconstructions of

40 to 45 x-y sections (0.4 µm each for FEMX-I cells, 0.2 µm for MSC; uncut) or 1-3 sections (section, green slice) are shown. The areas (dashed squares) covering SUN2-labeled nuclear envelope invaginations (dotted line) without Rab5a (A) or GALNT2 (B) are displayed in a single fluorescent channel. Percentage of FEMX-I cells with SUN2-stained nuclear envelope invaginations containing late (Rab7) or early (Rab5a) endosomes or without is indicated in the bar graph (C, >50 cells were evaluated per experiment, n = 3). **D, E**. FEMX-I cells were double-infected with baculovirus encoding for Rab7-RFP with either ER (D) or mitochondrial (E, Mito) marker–GFP fusion proteins, prior to their immunolabeling for SUN2 (purple). Cells were analyzed by CLSM. The relevant areas (dashed squares) are enlarged. Note the presence of ER luminal marker in nuclear membrane invagination (D, green arrowhead). To detect a weak signal of mitochondria into nuclear envelope invagination, the non-modified exposure of the GFP fluorescence was manually increased using Photoshop software (Adobe Photoshop CS6) (E, inset with zigzag arrow). Scale bars, 5 µm.

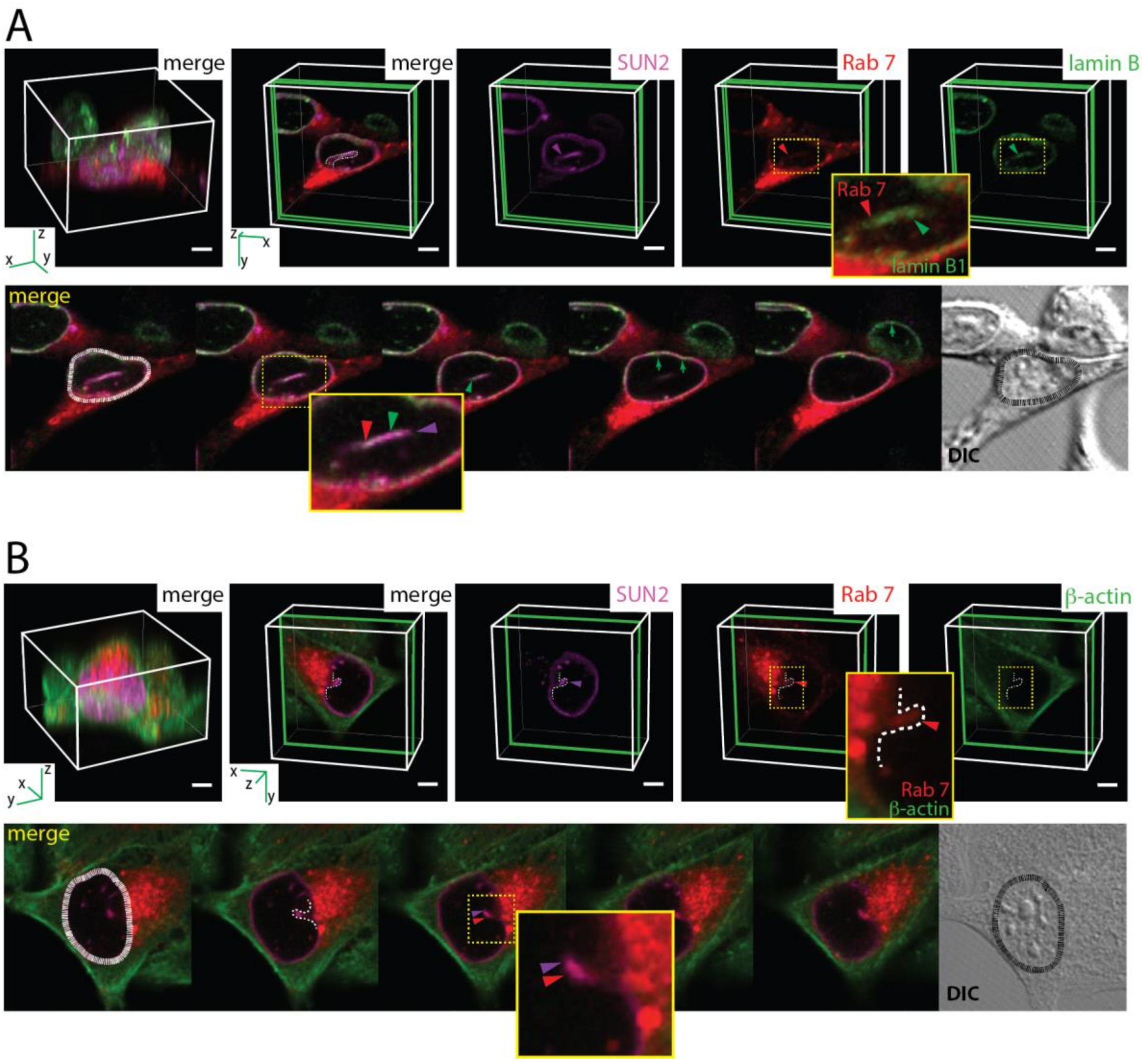

Supplementary Figure 3: Lamin B1 surrounds N-ALE. **A, B**. FEMX-I cells expressing Rab7–RFP were double-immunolabeled for SUN2 (purple) and lamin B1 (A, green) or β-actin (B, green) and analyzed by CLSM. 3D reconstructions of 40 to 45 x-y sections (0.4-µm each) (left top panel) or 1-3 sections (right top panels, green slice) are shown with the relevant individual x-y sections (bottom panels). A differential interference image (DIC) highlighting the position of nucleus (dotted line) is shown. The areas (dashed squares) covering SUN2-labeled nuclear envelope invaginations (dashed line, purple arrowhead) containing Rab7 (red arrowhead) are enlarged. Note the presence of lamin B1 (A, green arrowhead), but not β-actin (B) in the surrounding of N-ALE. Lamin B1 is also concentrated around nuclear membrane (A, green arrow). Scale bars, 5 µm.

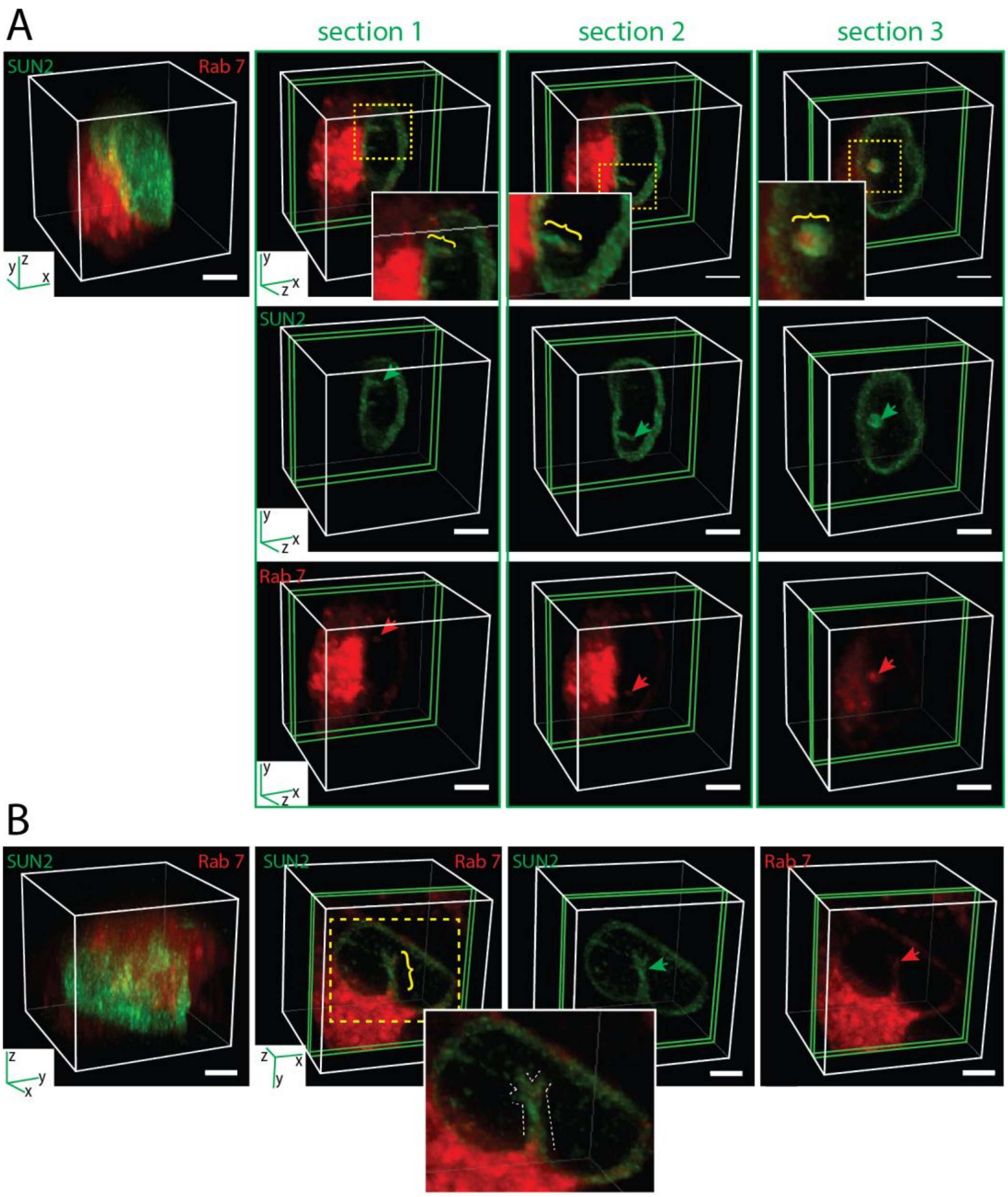

Supplementary Figure 4: Multiple N-ALE in FEMX-I melanoma cells. A, B. FEMX-I cells were double-labeled for SUN2 (green) and Rab7 (red) and analyzed by CLSM. 3D reconstructions of 40 to 45 x-y sections (0.4-µm each, left panels) or 1-3 sections (section, green slice) are shown. The areas (dashed square) covering SUN2-labeled nuclear envelope invaginations (green arrow) containing Rab7^{+} structures (red arrow) are enlarged. The merge and individual channels are presented. Note three distinct N-ALE (bracket, section 1, 2 and 3) in a single cell (A) or a branching one (B, dotted line). Scale bars, 5 µm.

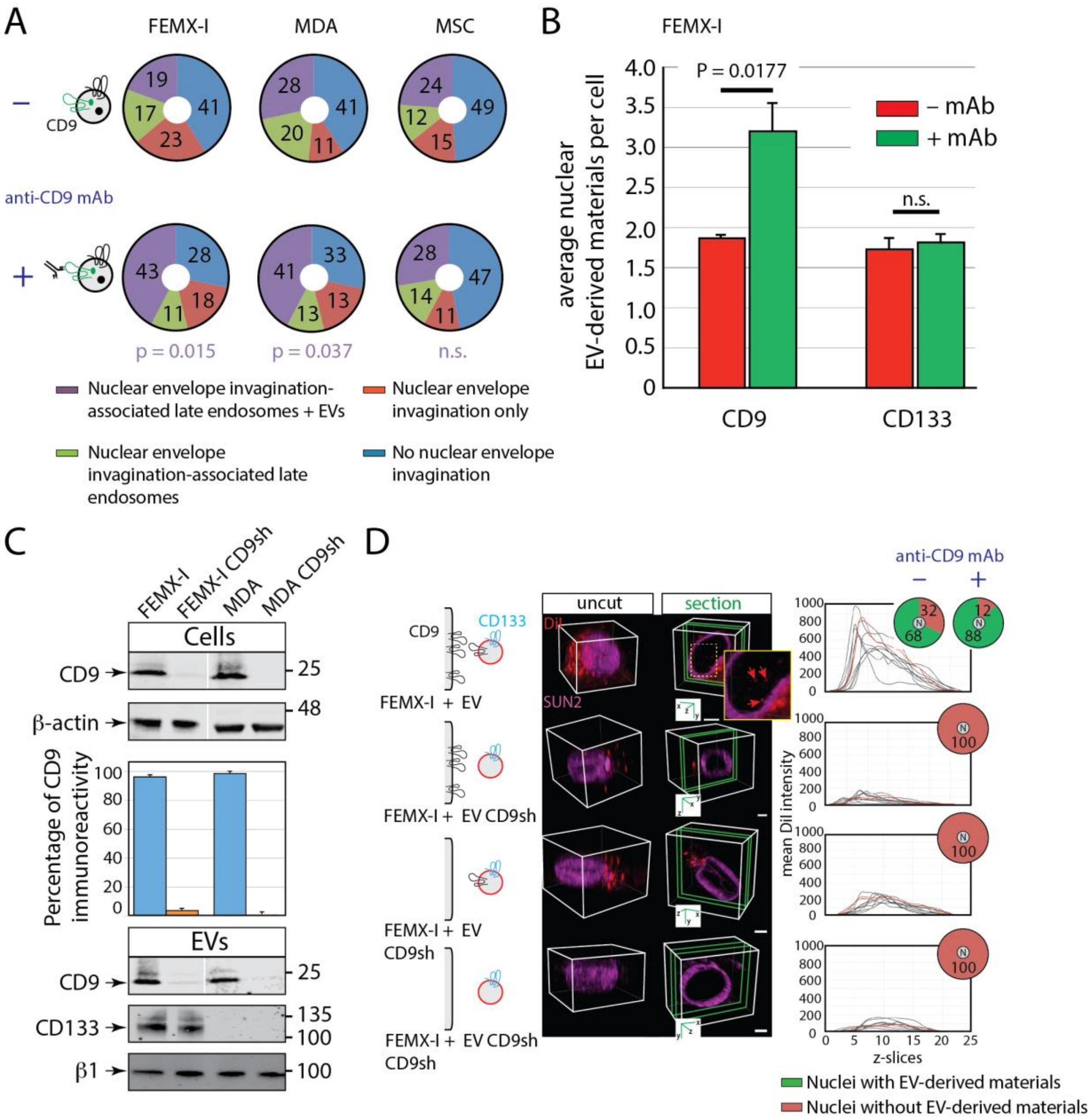

Supplementary Figure 5: The Endocytosis of EVs and their Transport to the Nucleoplasm Rely on the Tetraspanin Protein CD9. A. FEMX-I, MDA and MSCs expressing Rab7–RFP were incubated (4.5 h) with CD9-GFP^{+} EVs (5 x 10^{7} particles/ml) and monoclonal (m) Ab against CD9 (25 µg/ml) was added (+) or not (–) during the last 1.5 h prior to immunolabeling for SUN2, and analyzed by CLSM. Percentages of cells without or with SUN2-stained nuclear envelope invaginations containing or not Rab7–RFP (late endosomes) and CD9-GFP (+ EV) are indicated in pie charts (extended data are presented in Supplementary Table 1). The changes in the percentage of FEMX-I and MDA cells containing N-ALE + EVs upon incubation with anti-CD9 mAb are significantly different. N.s., not significant. **B**. Numbers of EV-derived biomaterials in the nucleus of recipient FEMX-I cells upon their exposure to CD9-GFP^{+} EVs in absence (–) or presence (+) of mAb

against CD9 or CD133 (mean ± s.e.m., >50 cells were evaluated per experiment, n = 3). **C**. Parental or CD9-depleted FEMX-I CD9sh and MDA CD9sh cells (top panels) and EVs released therefrom (bottom panels) were probed by immunoblotting for CD9, CD133 or importin β1. The reduction of CD9 expression was quantified using β-actin as an internal loading control (n = 3). MDA cells did not express CD133. Neither the latter nor importin β1 were upregulated upon the CD9 knockdown (for the blot source data, see Supplementary Figure 9). **D**. FEMX-I cells and DiI-labeled CD133$^+$ EVs expressing or not CD9 as indicated in the cartoons were incubated (4.5 h), immunostained for SUN2 (purple) and analyzed by CLSM. 3D reconstructions of 40-45 x-y sections (uncut) or 3 sections (section) of one cell are displayed. DiI-labeled biomaterials in the recipient cell nucleus are indicated (arrows), while total DiI intensity within the entire cell was quantified using ImageJ program. Percentage of cells with DiI-labeled EV-derived biomaterials in their nuclei (N) is indicated in pie charts. The addition of anti-CD9 mAb neither stimulated the endocytosis of EVs nor nuclear entry of EV-derived biomaterials when cells and/or EVs lacking CD9 were used. Scale bars, 5 µm.

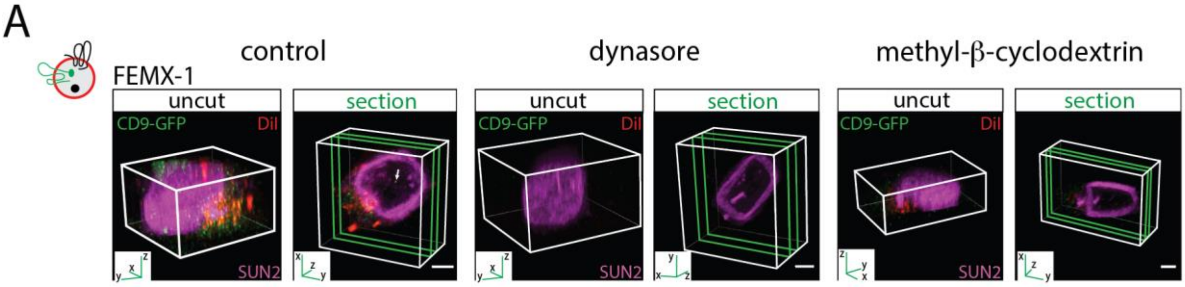

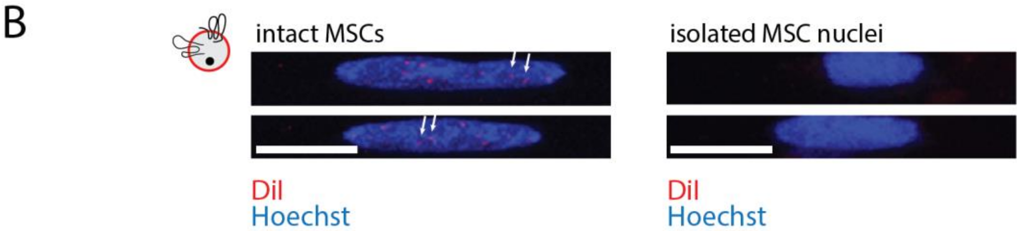

Supplementary Figure 6: Endocytosis is a Pre-Requisite for the Transport of EV-derived Biomaterials in the Nucleoplasm of Recipient Cells. A. FEMX-I cells were pre-treated (30 min) with dynasore or methyl-β–cyclodextrin and then incubated (4.5 h) with DiI-labeled CD133$^+$CD9-GFP$^+$ EVs (5 x 10^7 particles/ml). Cells were immunolabeled for SUN2 (purple) and analyzed by CLSM. DMSO was added as vehicle control (control). 3D reconstructions of 40 to 45 x-y sections (0.4-µm each, uncut) or 1-3 sections (green slice) are shown. Note the absence or strong reduction of EV-derived biomaterials in cells incubated

with dynasore or methyl-β–cyclodextrin, respectively. **B**. Intact MSCs or isolated nuclei were incubated with FEMX-I-derived DiI-labeled CD133^{+} EVs, and stained with Hoechst 33342 during the last 30 min. Composite z sections are shown. EV-derived biomaterials in the nucleus of recipient cell are indicated with arrows. Scale bars, 5 μm.

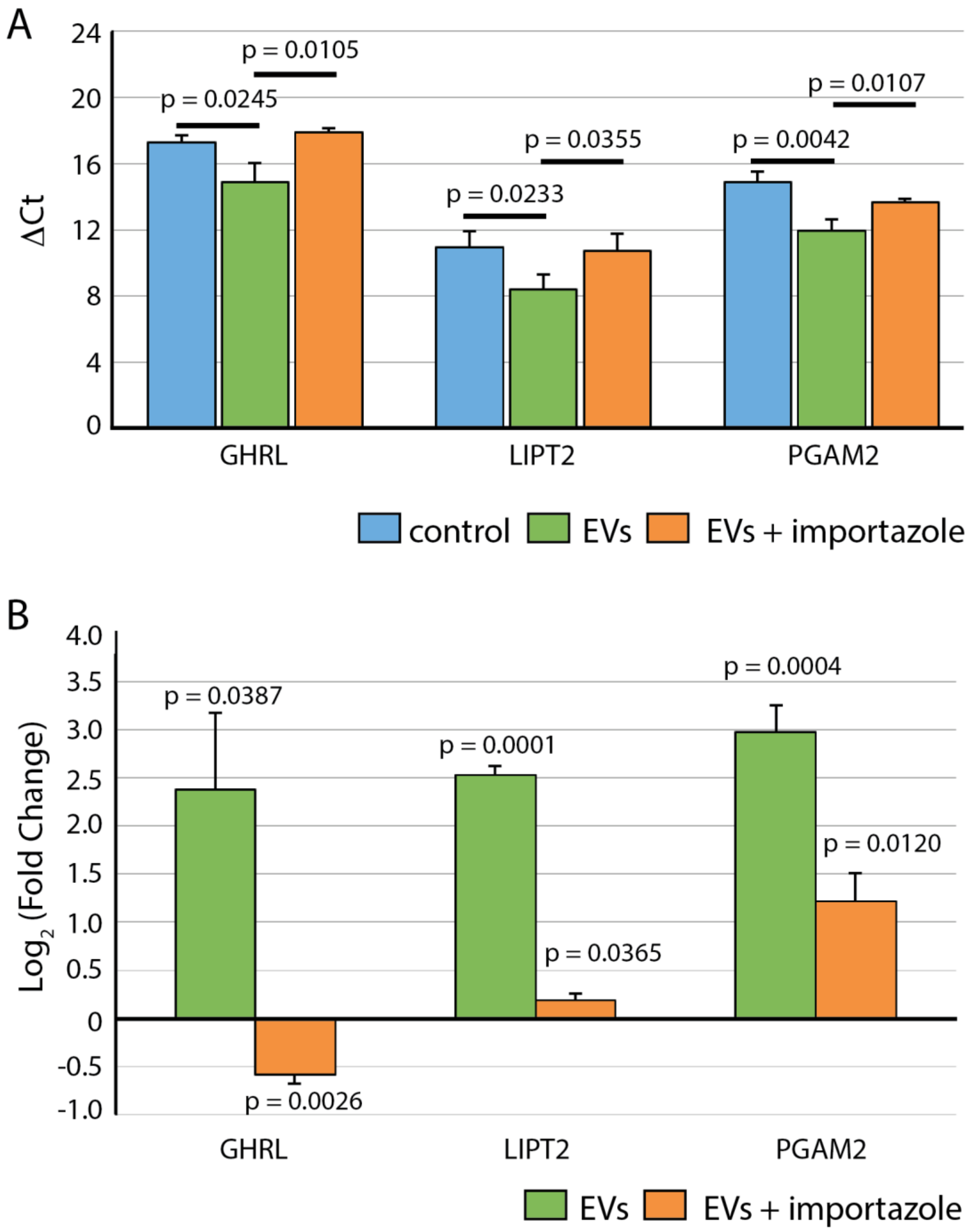

Supplementary Figure 7: Three MSC-associated Genes are Upregulated upon Exposure to Cancer Cell-derived EVs in the Absence of Importazole. A. Total RNA from MSCs exposed to solvent alone (control), 1 x 10^9 EVs derived from FEMX-I cells (EVs), or EVs in the presence of importazole (EVs + importazole) were analyzed by qRT-PCR. ΔC_T values for the genes of interest (GHRL, LIPT2, and PGAM2) were normalized to GAPDH expression (n = 3 independent experiments). **B**. Fold-changes in gene expression compared to control MSCs. Note the significant increase of GHRL, LIPT2, and PGAM2 transcripts in cells exposed to EVs by comparison to the minor change in the presence of importazole. Unpaired Student's t test was used. P values are indicated.

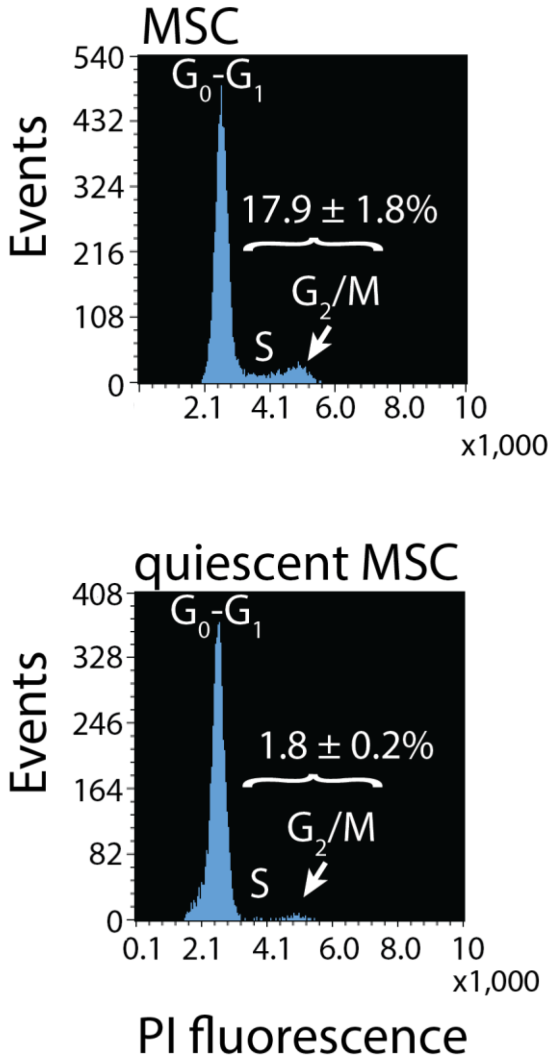

Supplementary Figure 8: Analysis of Quiescent MSCs by Flow Cytometry. Cell cycle phases of proliferating (top panel) and quiescent (bottom panel) MSCs were observed using propidium iodide (PI) staining and analyzed by flow cytometry. Percentage of cells in S and G_2/M phases is indicated.

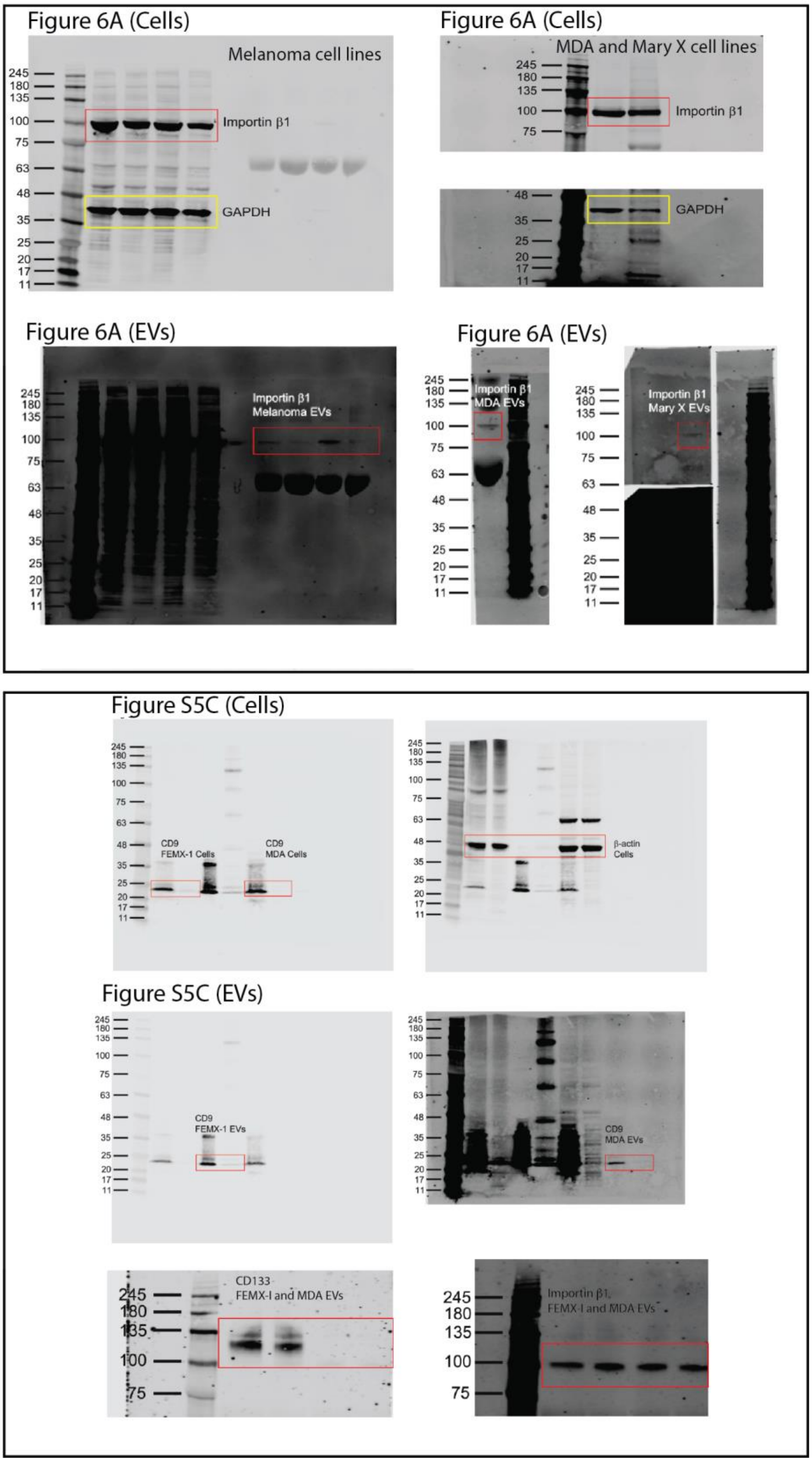

Supplementary Figure 9: Scans of Uncropped Immunoblots Shown in Figure 6 and Supplementary Figure 5. The relevant information presented in Figure 6 and Supplementary Figure 5 is indicated in red or yellow boxes. The molecular weight markers are presented on the left.

Supplemental Videos

Supplementary Video 1: CD9-GFP$^+$ EV-derived Biomaterials Localized in N-ALE, part I. The video (format: mp4; size: 2.5 MB) depicts a FEMX-I cell expressing Rab7-FRP (red) incubated with CD9-GFP$^+$ EVs (green) prior to immunolabeling with SUN2 Ab (purple). Cell was analyzed by CLSM. A 3D reconstruction with cutting plane (green slice) is displayed. Still images from this movie are shown in Figure 3C.

Supplementary Video 2: CD9-GFP$^+$ EV-derived Biomaterials Localized in N-ALE, part II. The video (format: mp4; size: 3.5 MB) depicts a FEMX-I cell incubated with CD9-GFP$^+$ EVs (green) prior to immunolabeling with SUN2 (purple) and Rab7 (red) Abs. Cell was analyzed by CLSM. A single slice (green) from a 3D reconstruction is displayed. Still image from this movie is shown in Figure 3F.

Supplementary Video 3: Subdomains of Late Endosomes Invading Nuclear Envelope Invaginations. The video (format: mp4; size: 2.9 MB) depicts a FEMX-I cell expressing Rab7-FRP (red) immunolabeled with SUN2 Ab (purple). Cell was analyzed by CLSM. 3D reconstruction of 3 sections (green slice) is displayed. Still image from this movie is shown in Figure 4A.

Supplemental Tables

Supplementary Table 1. Details of data presented in Figures 4, 5, 6, 8, and Supplementary Figure 5

Figure 4E

MSCs	Mean	SD	SEM
No nuclear membrane invagination	48.22%	1.37%	0.79%
Nuclear membrane invagination only	16.44%	0.31%	0.18%
Nuclear membrane invagination-associated late endosomes	35.33%	1.44%	0.83%

FEMX-I cells	Mean	SD	SEM
No nuclear membrane invagination	39.16%	7.67%	4.43%
Nuclear membrane invagination only	27.24%	3.44%	1.99%
Nuclear membrane invagination-associated late endosomes	33.60%	8.17%	4.72%

MDA cells	Mean	SD	SEM
No nuclear membrane invagination	39.03%	4.31%	2.49%
Nuclear membrane invagination only	18.19%	2.55%	1.47%
Nuclear membrane invagination-associated late endosomes	42.78%	2.83%	1.64%

Figure 4F

MSCs	Mean	SD	SEM
No nuclear membrane invagination	39.41%	7.70%	4.44%
Nuclear membrane invagination only	24.75%	2.41%	1.39%
Nuclear membrane invagination-associated late endosomes	35.84%	7.67%	4.43%

FEMX-I cells	Mean	SD	SEM
No nuclear membrane invagination	29.70%	5.60%	3.23%
Nuclear membrane invagination only	32.24%	5.63%	3.25%
Nuclear membrane invagination-associated late endosomes	38.06%	8.18%	4.72%

Figure 5C

	FEMX-I + 5 x 10^7 EVs				FEMX-I + 1 x 10^9 EVs		
	Mean	SD	SEM		Mean	SD	SEM
No nuclear membrane invagination	41.09%	4.96%	2.86%		17.78%	1.57%	0.91%
Nuclear membrane invagination only	22.70%	7.18%	4.14%		10.00%	4.71%	2.72%
Nuclear membrane invagination-associated late endosomes	16.77%	5.93%	3.42%		7.78%	1.57%	0.91%
Nuclear membrane invagination-associated late endosomes + EVs	19.44%	4.40%	2.54%		64.44%	4.16%	2.40%

Figure 6D

DMSO	Mean	SD	SEM
Nuclear membrane invagination without EV-derived materials	80%	6%	3%
Nuclear membrane invagination with EV-derived materials	20%	6%	3%
Nuclei without EV-derived materials	40%	4%	2%
Nuclei with EV-derived materials	60%	4%	2%

Importazole	Mean	SD	SEM
Nuclear membrane invagination without EV-derived materials	100%	0%	0%
Nuclear membrane invagination with EV-derived materials	0%	0%	0%
Nuclei without EV-derived materials	100%	0%	0%
Nuclei with EV-derived materials	0%	0%	0%

Figure 6E

Control	Mean	SD	SEM
Nuclei without EV-derived materials	33.33%	2.72%	1.57%
Nuclei with EV-derived materials	66.67%	2.72%	1.57%

Leptomycin B	Mean	SD	SEM
Nuclei without EV-derived materials	14.44%	1.57%	0.91%
Nuclei with EV-derived materials	85.56%	1.57%	0.91%

Figure 8B

	MSC				qMSC		
	Mean	SD	SEM		Mean	SD	SEM
No nuclear membrane invagination	48.96%	3.09%	1.79%		43.22%	2.73%	1.57%
Nuclear membrane invagination only	15.28%	1.96%	1.13%		13.22%	0.16%	0.09%
Nuclear membrane invagination-associated late endosomes	11.88%	3.80%	2.19%		21.78%	4.09%	2.36%
Nuclear membrane invagination-associated late endosomes + EVs	23.89%	0.79%	0.45%		21.78%	6.81%	3.93%

Figure 8C

MSC	– CD9 mAb				+CD9 mAb		
	Mean	SD	SEM		Mean	SD	SEM
Nuclei without EV-derived materials	44.65%	2.85%	1.64%		31.11%	5.67%	3.27%
Nuclei with EV-derived materials	55.35%	2.85%	1.64%		68.89%	5.67%	3.27%
qMSC							
Nuclei without EV-derived materials	100%	0%	0%		100%	0%	0%
Nuclei with EV-derived materials	0%	0%	0%		0%	0%	0%

Figure 8D and data not presented

MSC	Leptomycin B				Control		
	Mean	SD	SEM		Mean	SD	SEM
Nuclei without EV-derived materials	21.11%	1.57%	0.91%		43.65%	3.02%	2.14%
Nuclei with EV-derived materials	78.89%	1.57%	0.91%		56.35%	3.02%	2.14%
qMSC							
Nuclei without EV-derived materials	100%	0%	0%		100%	0%	0%
Nuclei with EV-derived materials	0%	0%	0%		0%	0%	0%

Supplementary Figure 5A

FEMX-I cells	– CD9 mAb				+ CD9 mAb		
	Mean	SD	SEM	P value	Mean	SD	SEM
No nuclear membrane invagination	41.09%	4.96%	2.86%		28.21%	8.55%	4.94%
Nuclear membrane invagination only	22.70%	7.18%	4.14%		18.03%	9.89%	5.71%
Nuclear membrane invagination-associated late endosomes	16.77%	5.93%	3.42%		11.37%	4.10%	2.37%
Nuclear membrane invagination-associated late endosomes + EVs	19.44%	4.40%	2.54%	0.015	42.39%	6.04%	3.49%

MDA cells	– CD9 mAb				+ CD9 mAb		
	Mean	SD	SEM	P value	Mean	SD	SEM
No nuclear membrane invagination	40.56%	3.42%	1.98%		33.33%	2.72%	1.57%
Nuclear membrane invagination only	11.11%	3.14%	1.81%		12.87%	2.18%	1.26%
Nuclear membrane invagination-associated late endosomes	20.00%	9.81%	5.67%		12.59%	1.05%	0.60%
Nuclear membrane invagination-associated late endosomes + EVs	28.33%	5.93%	3.42%	0.037	41.20%	4.13%	2.39%

MSCs	– CD9 mAb			P value	+ CD9 mAb		
	Mean	SD	SEM		Mean	SD	SEM
No nuclear membrane invagination	48.96%	3.09%	1.79%		46.67%	2.72%	1.57%
Nuclear membrane invagination only	15.28%	1.96%	1.13%		11.11%	1.57%	0.91%
Nuclear membrane invagination-associated late endosomes	11.88%	3.80%	2.19%		14.44%	6.85%	3.95%
Nuclear membrane invagination-associated late endosomes + EVs	23.89%	0.79%	0.45%	NS	27.78%	4.16%	2.40%

Supplementary Figure 5D

FEMX-I + EV	– CD9 mAb				+ CD9 mAb		
	Mean	SD	SEM		Mean	SD	SEM
Nuclei without EV-derived materials	32.02%	1.63%	0.94%		12.39%	1.71%	0.98%
Nuclei with EV-derived materials	67.98%	1.63%	0.94%		87.61%	1.71%	0.98%

FEMX-I + EV CD9sh<br>FEMX-I CD9sh + EV<br>FEMX-I CD9sh + EV CD9sh							
Nuclei without EV-derived materials	100%	0%	0%		100%	0%	0%
Nuclei with EV-derived materials	0%	0%	0%		0%	0%	0%

SD, standard deviation; SEM, standard error of the mean; NS, not significant.
>50 cells were evaluated per experiment, n = 3.
Color code refers to the pie chart presented in the figure.

Supplementary Table 2. Primary antibodies used for immunodetection and functional assay

Antigen	Clone/AS (host species)	Manufacturer	Dilution		
			ICC/IHC	IB	FA
CD9	HI9a (m)	Biolegend			1:20
	P1/33/2 (m)	Santa Cruz Biotechnology		1:500	
	72F6 (m)	Thermo Scientific	1:50		
SUN2	H-145 (r)	Santa Cruz Biotechnology	1:50		
	A-10 (m)	Santa Cruz Biotechnology	1:50		
Importin β1	H-7 (m)	Santa Cruz Biotechnology	1:50	1:500	
CD133	W6B3C1 (m)	Miltenyi Biotec	1:50	1:100	
Alix	1A12 (m)	Santa Cruz Biotechnology	1:50	1:200	
Annexin A2	3D5 (m)	Santa Cruz Biotechnology	1:50		
Nucleoporins	Mab414 (m)	Enzo Life Sciences	1:50		
β-Actin	C4 (m)	Santa Cruz Biotechnology		1:1000	
	GT5512 (m)	GeneTex	1:200		
GAPDH	686613 (m)	R&D Systems		1:10000	
Rab7	EPR7589 (r)	Abcam	1:50		
Rab7-Alexa Fluor 647	EPR7589 (r)	Abcam	1:100		
pan-Cytokeratin	H-240 (r)	Santa Cruz Biotechnology	1:50		
Vimentin	RV202 (m)	Abcam	1:100		
	EPR3776 (r)	Abcam	1:100		
Lamin B1	119D5-F1 (m)	Abcam	1:50		

AS, antiserum; ICC, immunocytochemistry; IHC, immunohistochemistry; IB, immunoblotting; FA, functional assay
M, mouse; R, rabbit
